# Supplementary material for: Dissecting and steering cell dynamics using spatially-informed RNA velocity with veloAgent
Source: Mol Syst Biol. 2026 May 6;22(7):1180–200. doi: 10.1038/s44320-026-00213-w (PMC13328566; doi:10.1038/s44320-026-00213-w)
Supplement: Supplementary file 2 — Appendix [file 44320_2026_213_MOESM2_ESM.pdf]

Appendix for Dissecting and steering cell dynamics using spatially informed RNA velocity with veloAgent

Table of Contents

Appendix Supplementary Figures 2

Appendix Figure S1 2

Appendix Figure S2 3

Appendix Figure S3 4

Appendix Figure S4 5

Appendix Figure S5 6

Appendix Figure S6 8

Appendix Figure S7 9

Appendix Figure S8 10

Appendix Figure S9 11

Appendix Figure S10 12

Appendix Figure S11 13

Appendix Figure S12 15

Appendix Figure S13 16

Appendix Figure S14 17

Appendix Figure S15 18

Appendix Figure S16 19

Appendix Figure S17 20

Appendix Figure S18 21

Appendix Figure S19 22

Appendix Figure S20 23

Appendix Supplementary Tables 24

Appendix Table S1 25

Appendix Table S2 26

Appendix Table S3 27

Appendix Table S4 28

Appendix Supplementary Figures

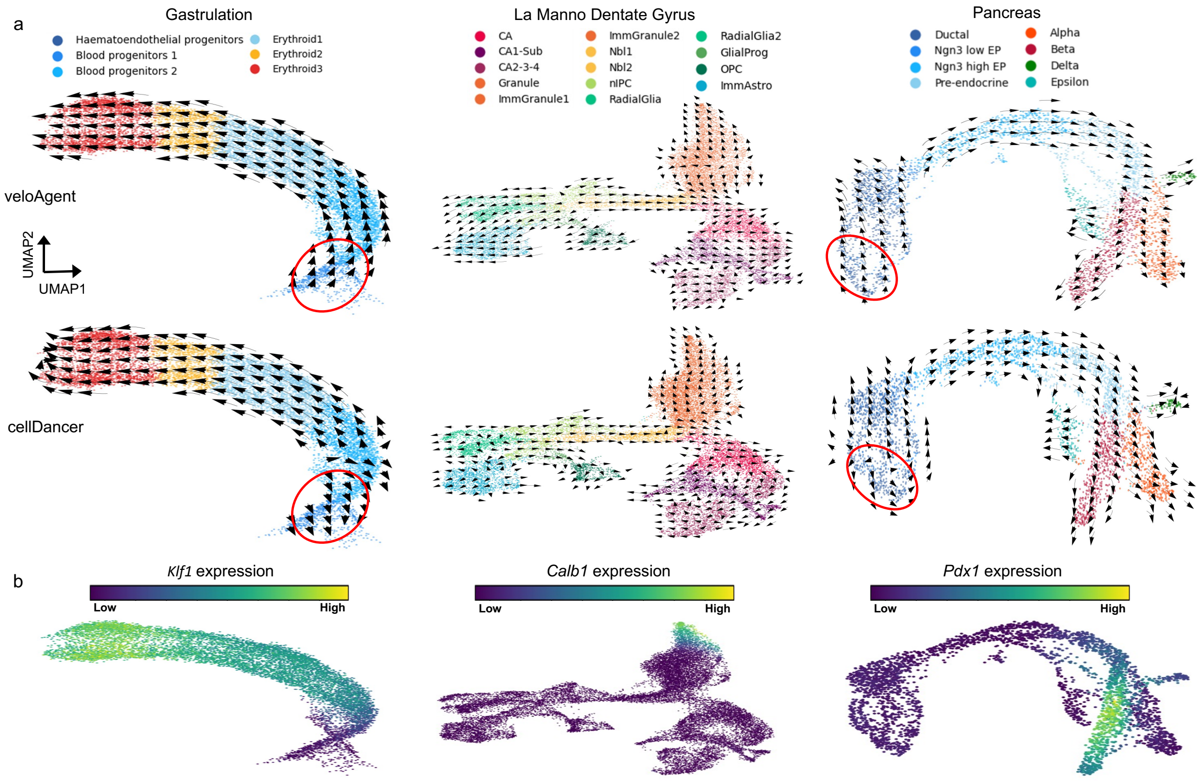

Appendix Figure S1: UMAP projections of veloAgent for scRNA-seq (non-spatial) RNA velocity inference. **a**, RNA velocity vectors computed by veloAgent (top) and cellDancer (bottom), projected onto UMAP embeddings of three datasets: gastrulation; dentate gyrus; and pancreas. Red circles highlight key regions of disagreement between methods. **b,** Expression levels of marker genes in terminal cell populations: *Klf1* in gastrulation (left), *Calb1* in dentate gyrus (middle), and *Pdx1* in pancreas (right).

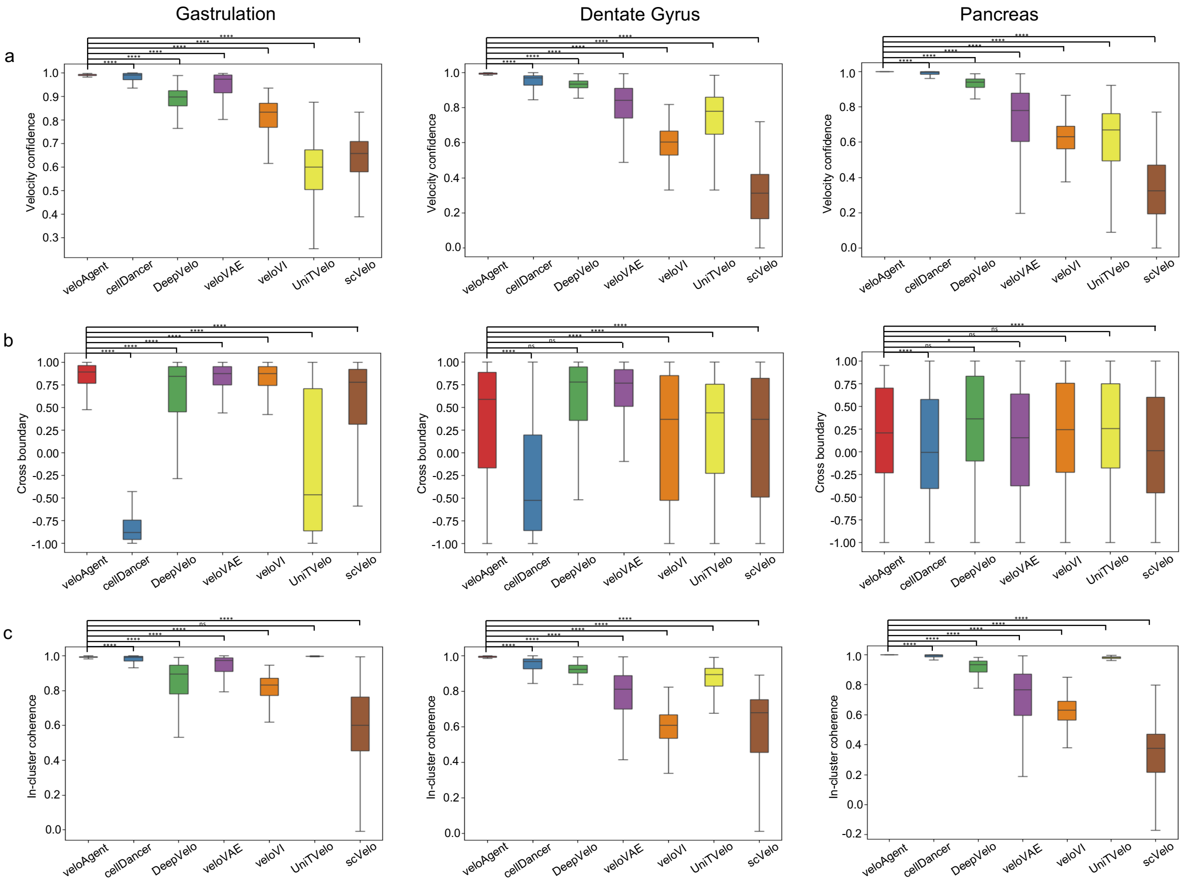

Appendix Figure S2: Quantitative performance benchmarking of veloAgent for scRNA-seq (non-spatial) RNA velocity inference. **a,** Velocity confidence scores across methods (veloAgent, cellDancer, DeepVelo, veloVAE, veloVI, UniTVelo, scVelo). **b,** Cross-boundary scores across methods. **c,** In-cluster coherence scores across methods. Error bars show interquartile range. Statistical significance calculated using Mann-Whitney U-test with FDR correction. Significance levels are indicated as follows: n.s, not significant ($P \geq 0.05$); * ($P < 0.05$); ** ($P < 0.01$); *** ($P < 0.001$); and **** ($P < 0.0001$). Exact *P*-values can be found in Appendix Table S2.

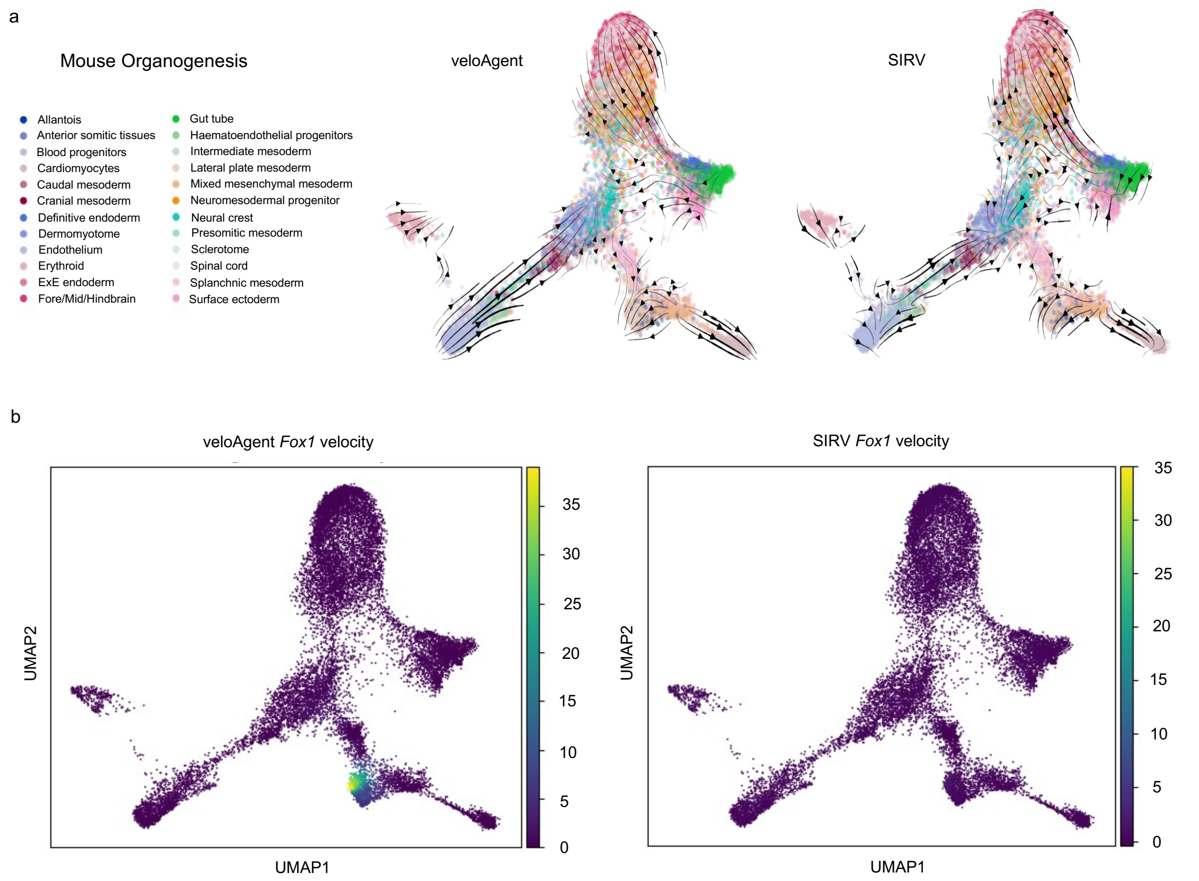

Appendix Figure S3: Evaluation of veloAgent's RNA velocity inference compared with SIRV on seq-FISH mouse organogenesis dataset. a, RNA velocity vectors inferred by veloAgent (left) and SIRV (right), projected onto UMAP embeddings of mouse organogenesis. **b,** Expression levels of *Foxf1*, a marker gene of the terminal cell state lateral plate mesoderm.

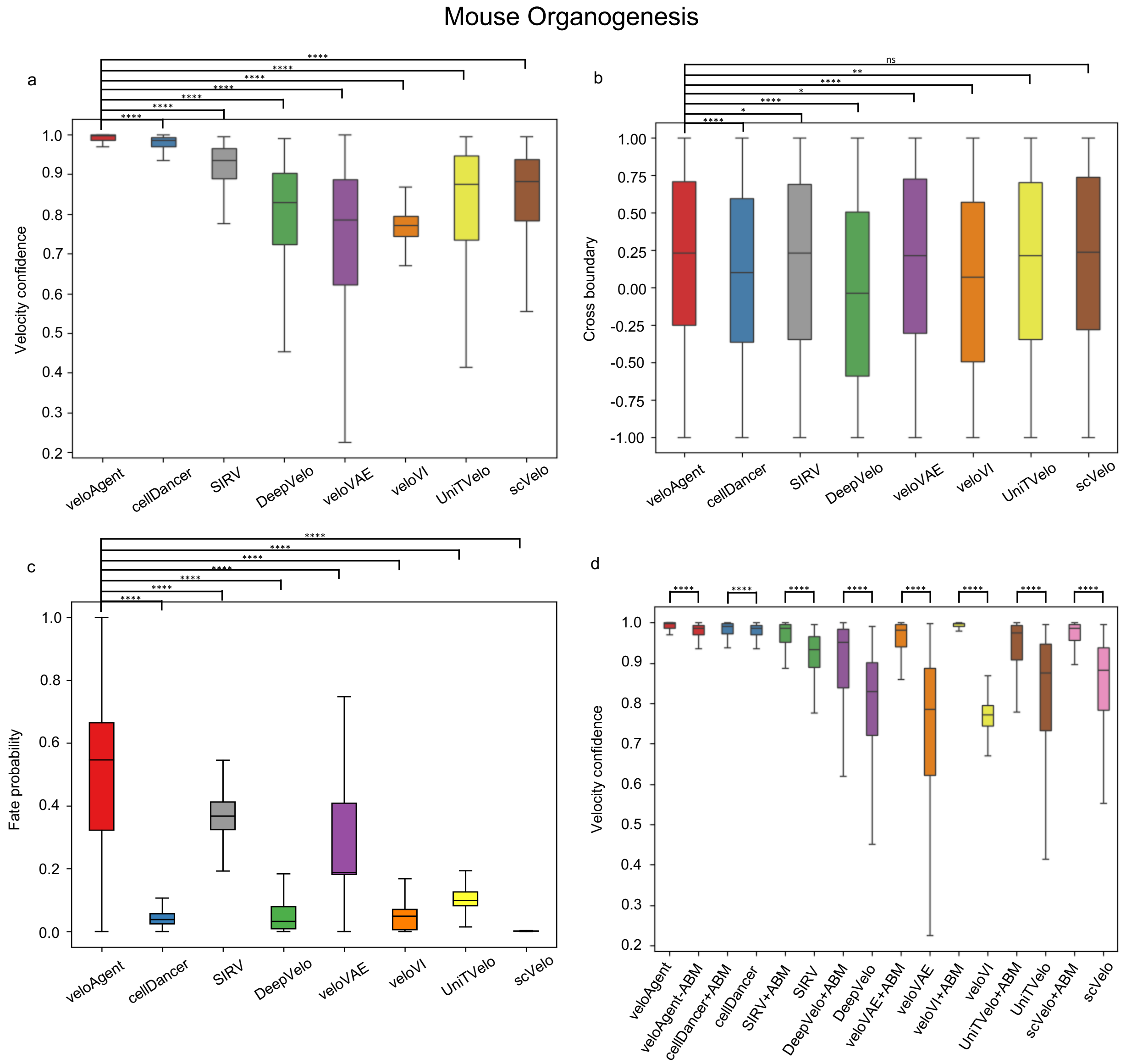

Appendix Figure S4: Quantitative performance benchmarking of veloAgent for the seq-FISH mouse organogenesis dataset. a, Velocity confidence scores across methods (veloAgent, CellDancer, DeepVelo, veloVAE, veloVI, UniTVelo, scVelo). **b,** Cross-boundary scores across methods. **c,** Fate probability scores across methods. **d,** Velocity confidence scores before and after applying veloAgent's agent-based model (ABM) for spatial refinement, contrasting original temporal velocity estimates of each method with their spatially adjusted counterparts using veloAgent's ABM. Error bars show interquartile range. Statistical significance calculated using Mann-Whitney U-test with FDR correction. Significance levels are indicated as follows: n.s, not significant ($P \geq 0.05$); * ($P < 0.05$); ** ($P < 0.01$); *** ($P < 0.001$); and **** ($P < 0.0001$).

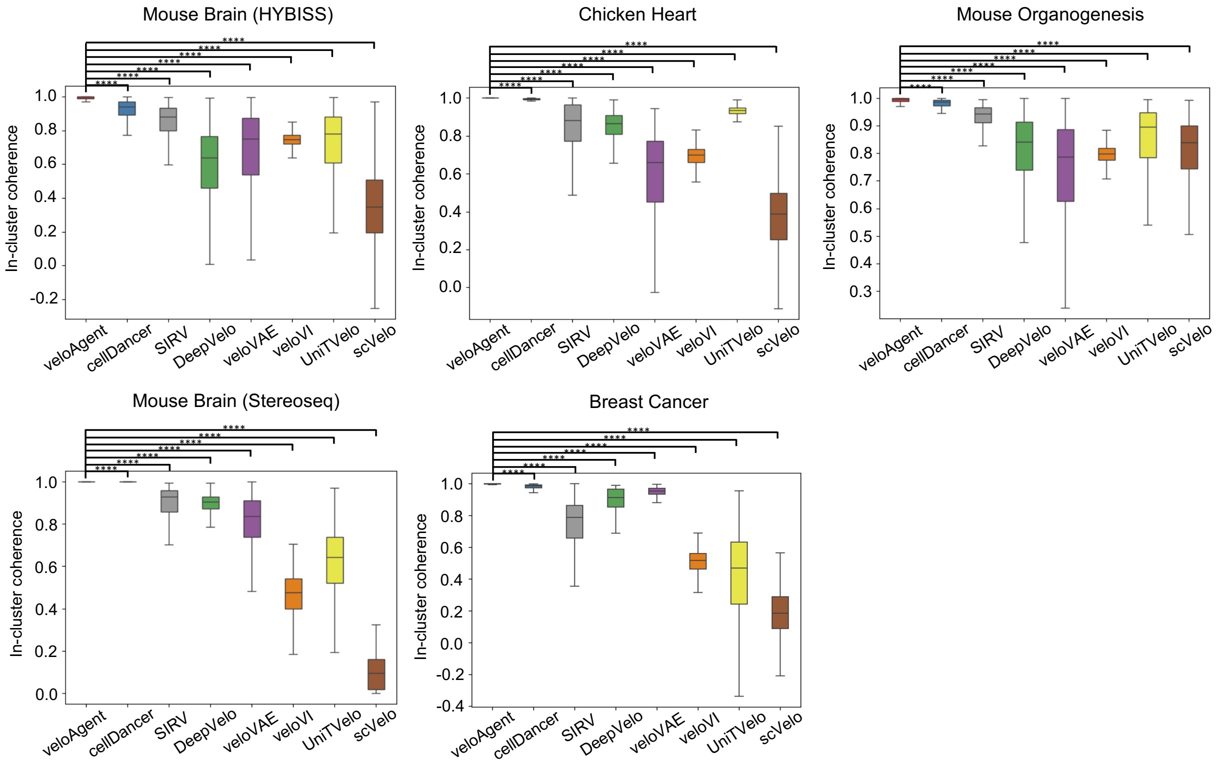

Appendix Figure S5: In-cluster coherence benchmarking of veloAgent. Comparison of in-cluster coherence scores between veloAgent and other RNA velocity models across five datasets: mouse brain (HybISS), chicken heart, mouse organogenesis, mouse brain (Stereo-seq), and breast cancer. Higher scores indicate greater consistency of velocities within clusters, with veloAgent showing superior performance. Error bars show interquartile range. Statistical significance calculated using Mann-Whitney U-test with FDR correction. Significance levels are indicated as follows: n.s, not significant ($P \geq 0.05$); * ($P < 0.05$); ** ($P < 0.01$); *** ($P < 0.001$); and **** ($P < 0.0001$).

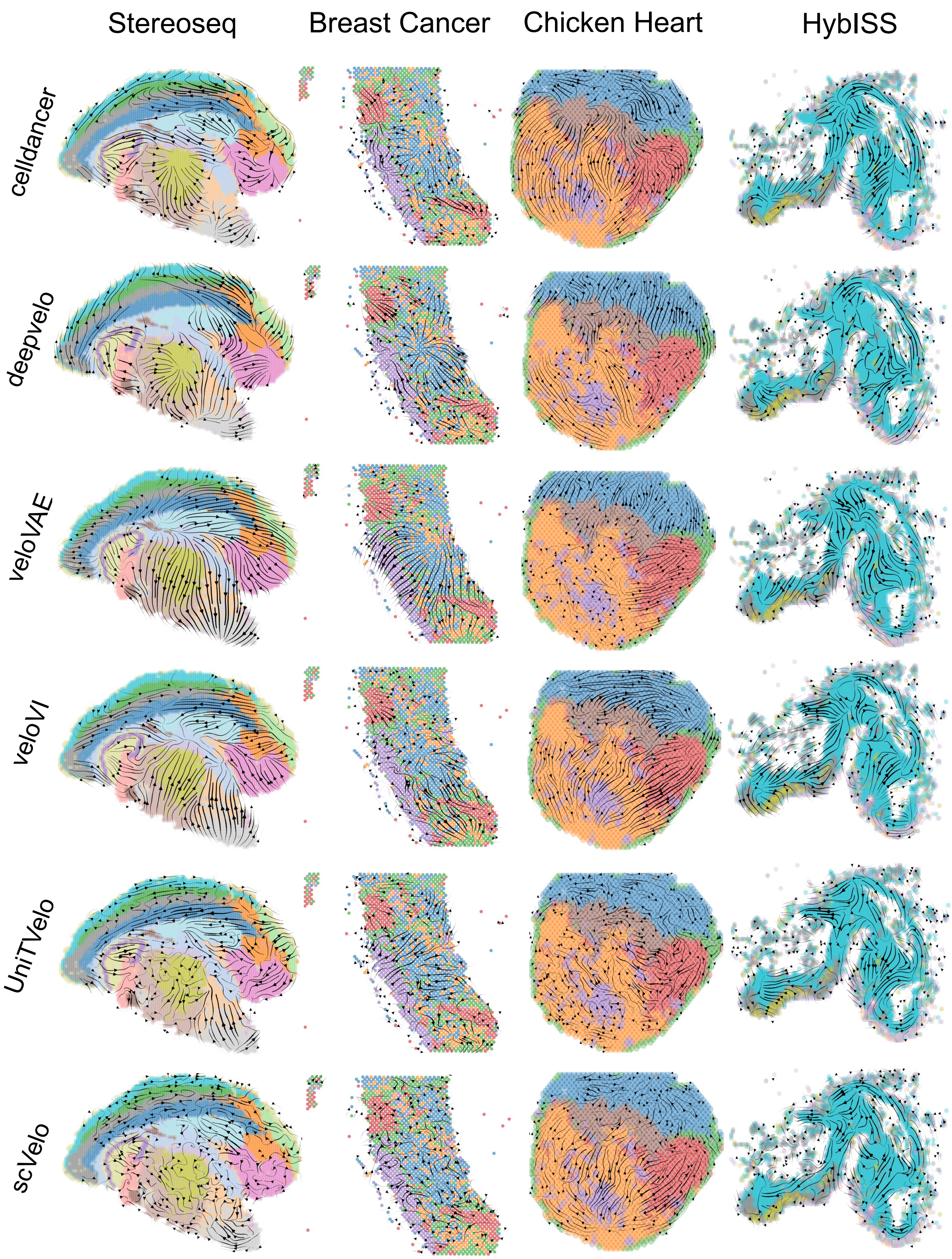

Stereoseq
Breast Cancer
Chicken Heart
HybISS
celldancer
deepvelo
veloVAE
veloVI
UniTVelo
scVelo

Appendix Figure S6: Spatial RNA velocity inference of state-of-the-art methods. Spatial RNA velocity fields computed by cellDancer, DeepVelo, veloVAE, veloVI, UniTVelo, scVelo overlaid on tissue coordinate maps for mouse brain (Stereo-seq), breast cancer, chicken heart; and mouse brain (HYBISS) datasets.

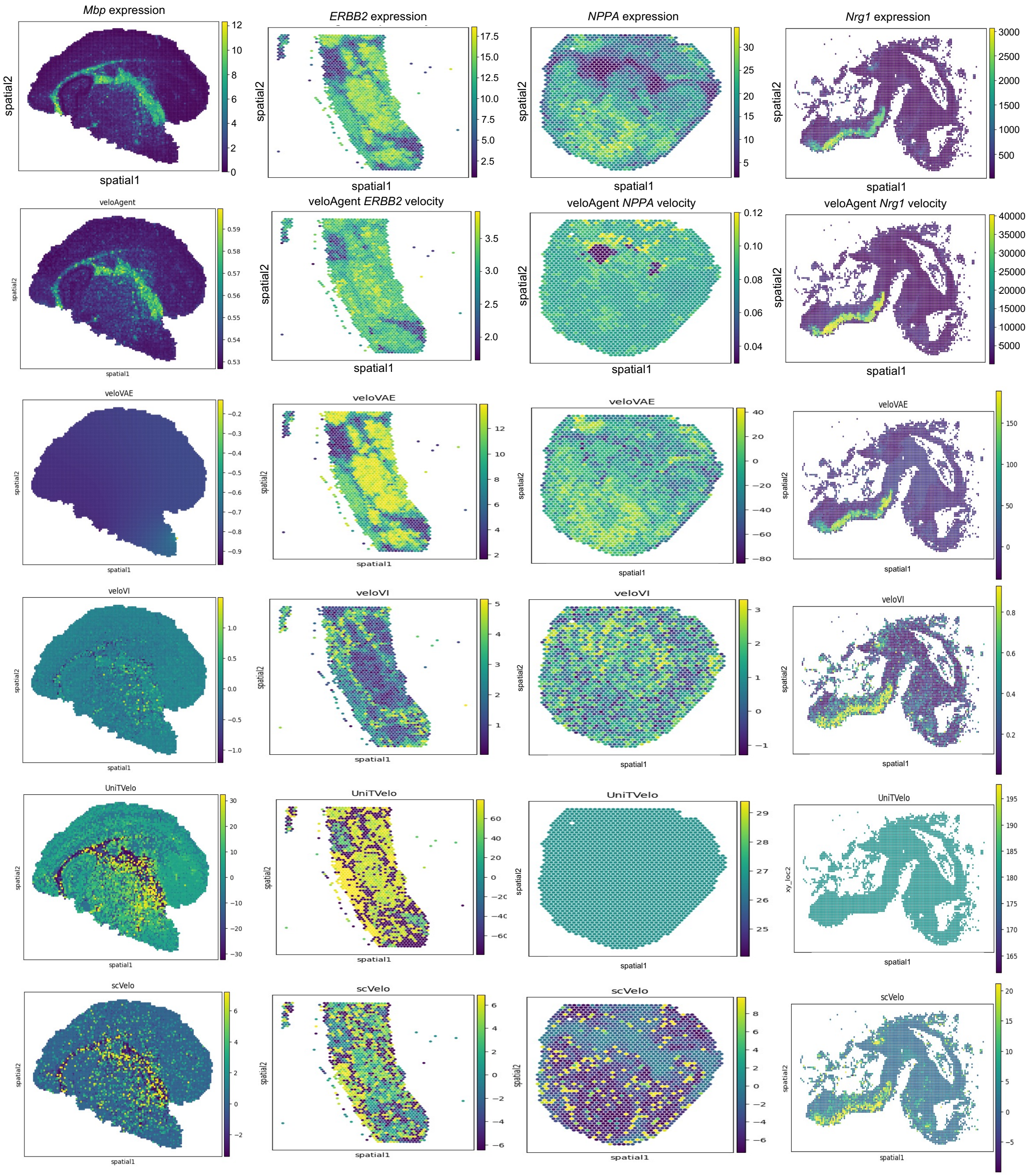

Appendix Figure S7: Spatial RNA velocity fields for the marker genes in each dataset, comparing veloAgent with veloVAE, veloVI, UniTVelo and scVelo. veloAgent captures clearer and more localized velocity patterns toward terminal regions, consistent with expected developmental trajectories.

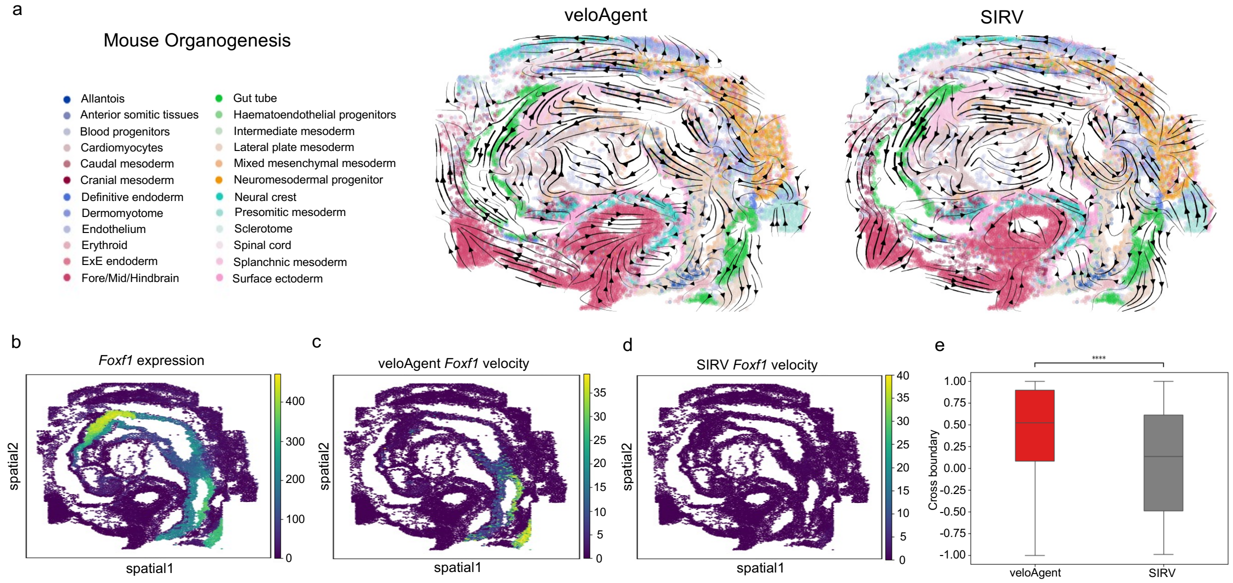

Appendix Figure S8: Evaluation of veloAgent's RNA velocity inference compared with SIRV on the **seq-FISH mouse organogenesis dataset on spatial maps. a,** RNA velocity vectors inferred by veloAgent (left) and SIRV (right), projected onto spatial embeddings of mouse organogenesis. **b,** Expression of *Foxf1*, a marker gene of the terminal cell state lateral plate mesoderm, on the spatial map. **c, d** Velocity fields of *Foxf1* in the lateral plate mesoderm, on the spatial velocity map, comparing **c,** veloAgent and **d,** SIRV (right). veloAgent demonstrates a clear increase in velocity within the terminal region, whereas SIRV shows indiscernible velocities. **e,** Cross-boundary scores of veloAgent and SIRV on the spatial map. Error bars show interquartile range. Statistical significance calculated using Mann-Whitney U-test with FDR correction. Significance levels are indicated as follows: n.s, not significant ($P \geq 0.05$); * ($P < 0.05$); ** ($P < 0.01$); *** ($P < 0.001$); and **** ($P < 0.0001$).

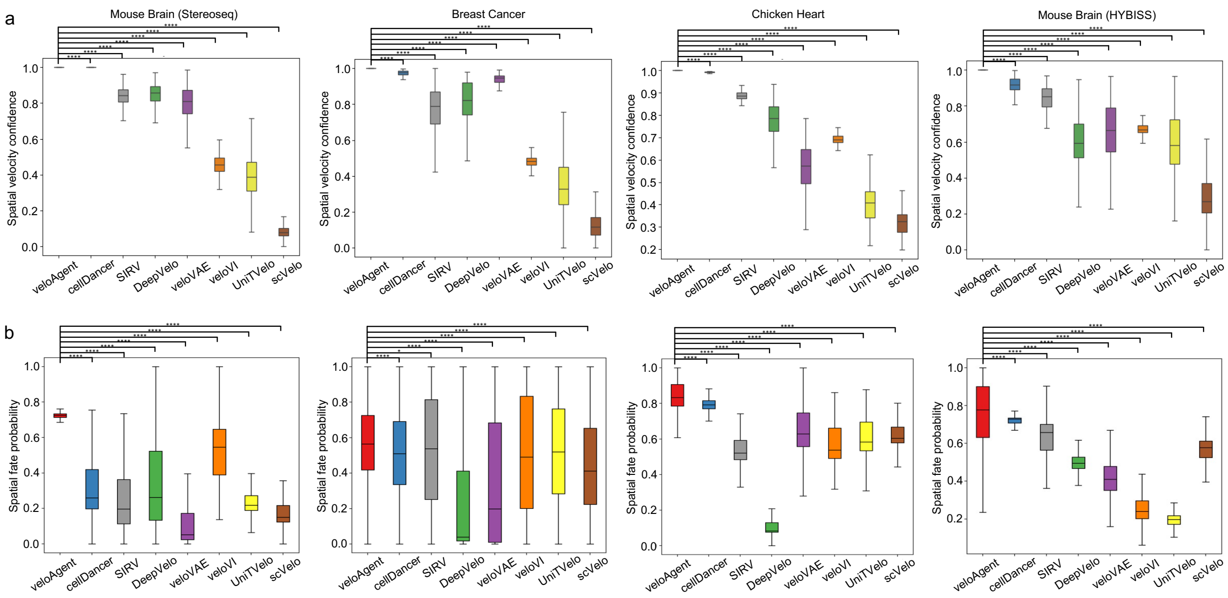

Appendix Figure S9: Evaluation of RNA velocity quality on spatial map. a, Spatial velocity confidence (mean velocity confidence across sub-grids of dataset) comparison between veloAgent and all other methods. **b,** Spatial fate probability (fate probability calculated based off spatial mapping) score evaluations between veloAgent and all other methods. veloAgent's performance gap from other methods and its consistency emphasizes the importance of spatial context in RNA velocity inference. Error bars show interquartile range. Statistical significance calculated using Mann-Whitney U-test with FDR correction. Significance levels are indicated as follows: n.s, not significant ($P \geq 0.05$); * ($P < 0.05$); ** ($P < 0.01$); *** ($P < 0.001$); and **** ($P < 0.0001$).

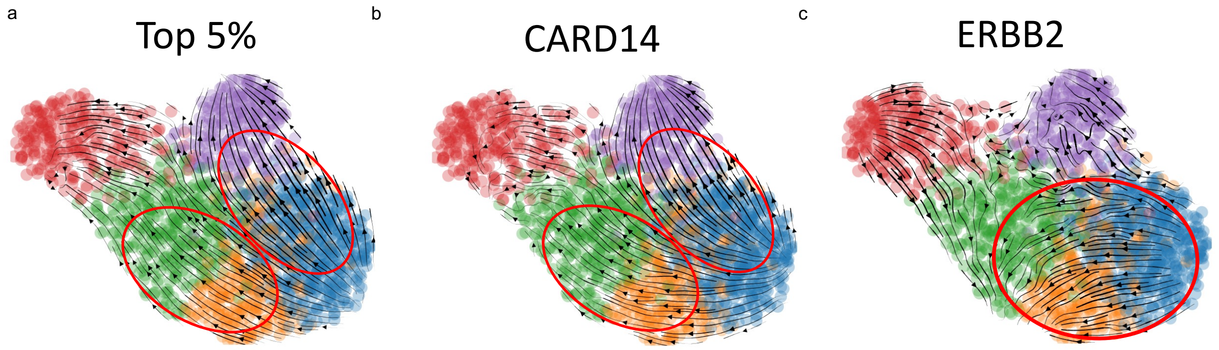

Appendix Figure S10: Single-gene perturbation analysis. UMAP projections show velocity perturbations for **a,** the top 5% most affected genes, **b,** *CARD14*, and **c,** *ERBB2*. Among the perturbed genes, *CARD14* and *ERBB2* emerged as the most strongly affected, exhibiting the largest trajectory reversals upon perturbation.

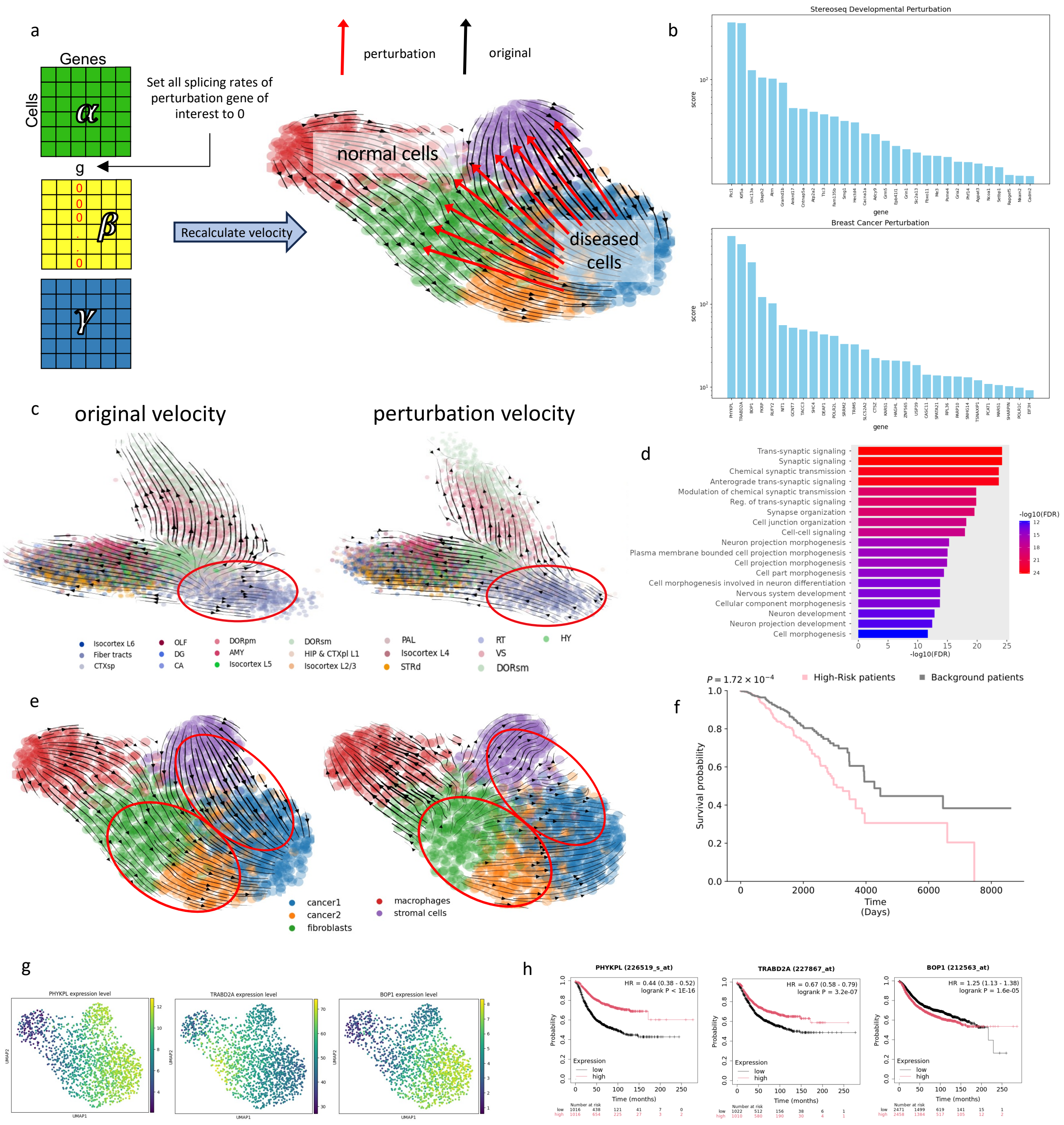

Appendix Figure S11: In-silico perturbation of RNA splicing rates (β) by veloAgent. veloAgent enables in-silico perturbation by systematically silencing splicing to assess the impact of gene-specific regulatory loss on cellular dynamics. **a,** Schematic of the perturbation pipeline, where the splicing rate (β) of each gene is individually set to zero to simulate gene knockout or drug-induced repression, followed by RNA velocity recalculation. **b,** Top 5% of perturbed genes ranked by the change in cross-boundary score of the velocity following perturbation in mouse brain developmental data (top) and human breast cancer data (bottom). **c,** Comparison of perturbed versus original velocity projections in the developmental dataset, with red circles highlighting regions of substantial deviation. **d,** GO enrichment analysis of the top 5% most perturbed genes, revealing biological processes associated with developmental regulation. **e,** Velocity projection

comparison in the breast cancer dataset showing notable shifts following splicing perturbation. **f,** Survival analysis comparing patient outcomes between high- and low-expression groups for the combined set of significantly perturbed genes in the breast cancer dataset. **g,** UMAP projections showing expression of the top three candidate therapeutic targets, *PHYKPL, TRABD2A* and *BOP1* each enriched in cancer-associated cell populations. **h,** Individual Kaplan-Meier survival analyses for *PHYKPL, TRABD2A* and *BOP1*, demonstrating prognostic relevance based on expression level.

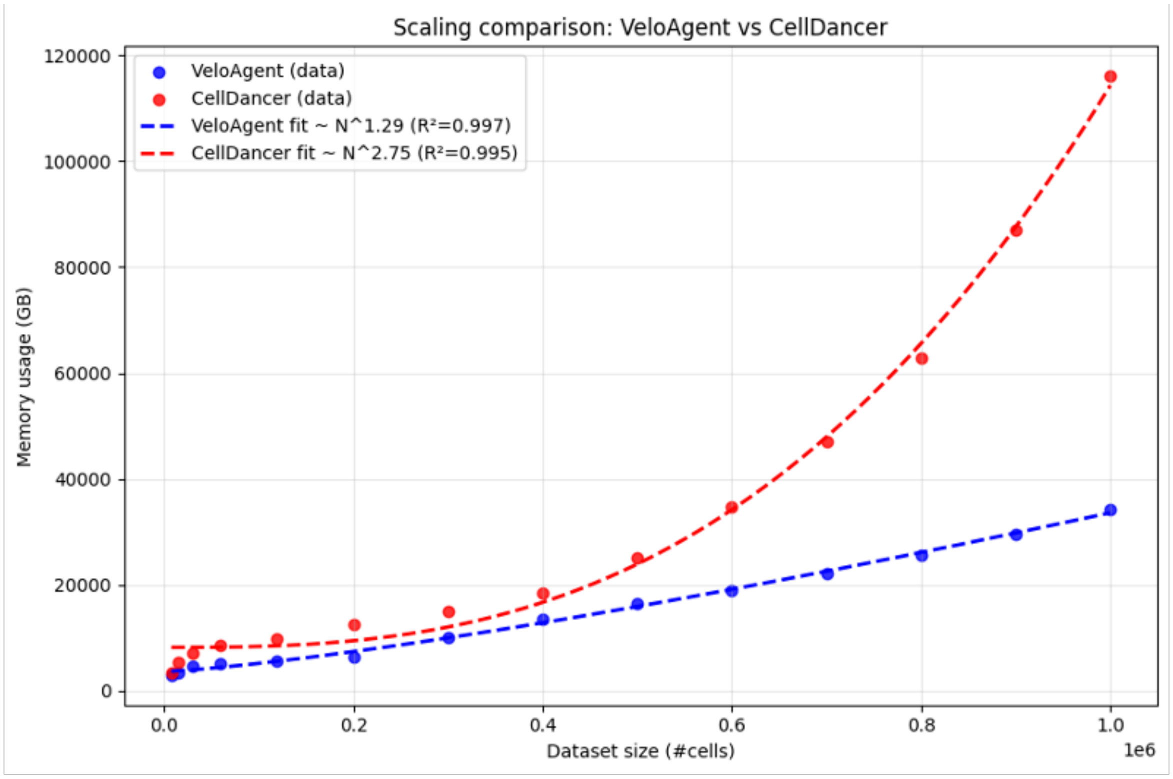

Appendix Figure S12: Memory scaling of veloAgent and cellDancer across increasing dataset sizes. Memory usage (GB) as a function of dataset size (number of cells) for veloAgent (blue) and cellDancer (red). The plot represents measured memory consumption across datasets ranging from 7,392 to 1,000,000 cells. Dashed lines denote curve fits: veloAgent scales approximately as $N^{1.29}$ ($R^2 = 0.997$), consistent with near-linear growth, while cellDancer scales approximately as $N^{2.75}$ ($R^2 = 0.995$), reflecting polynomial (near-cubic) growth. These results demonstrate that veloAgent maintains tractable memory requirements at scale, whereas cellDancer exhibits rapidly increasing memory usage, limiting its applicability to large datasets.

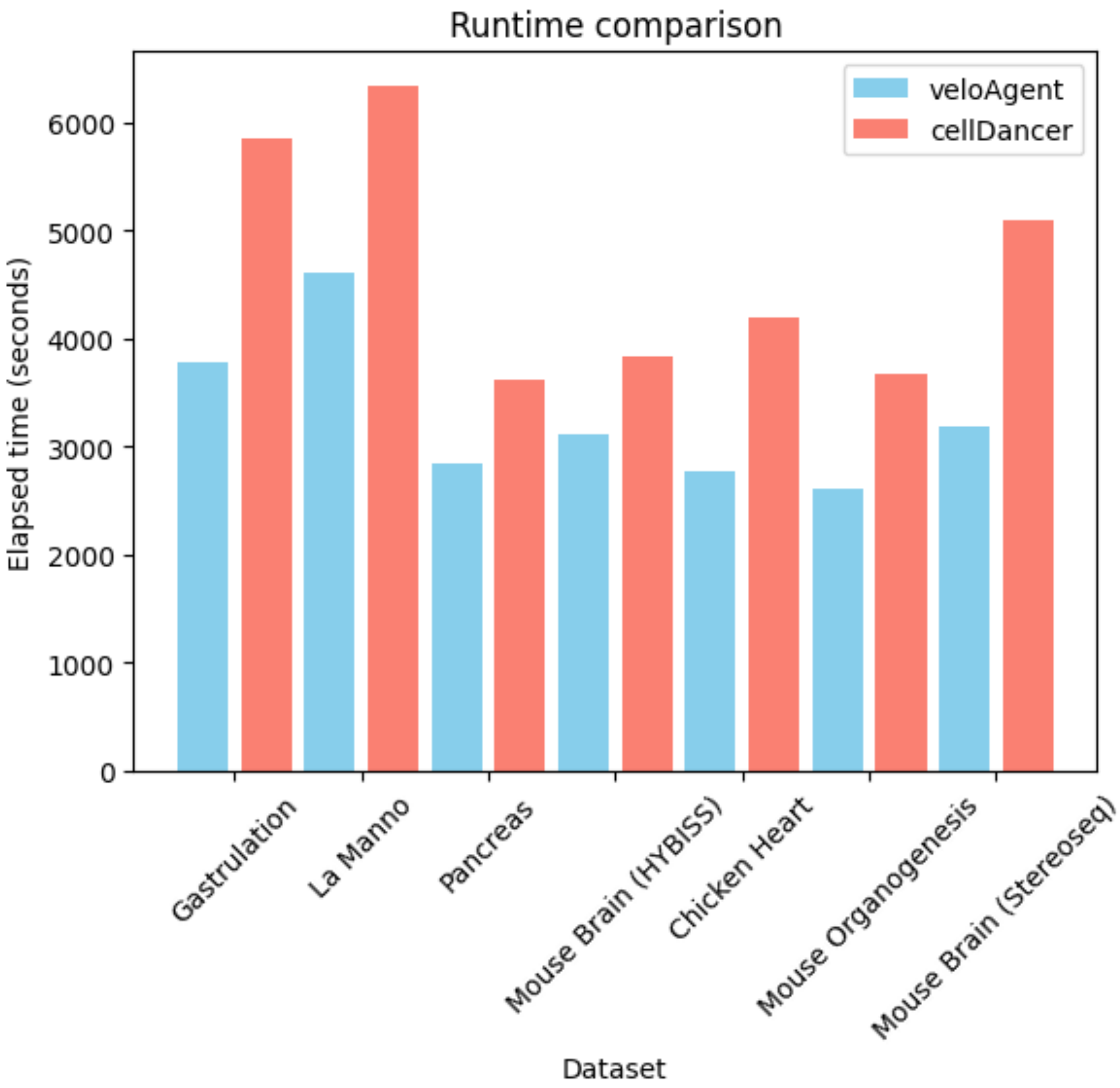

Appendix Figure S13: Runtime comparison of veloAgent and cellDancer. Elapsed runtimes of veloAgent and CellDancer across scRNA-seq and spatial transcriptomics datasets. veloAgent consistently demonstrates shorter runtimes than CellDancer, highlighting its scalability across datasets of varying size and modality.

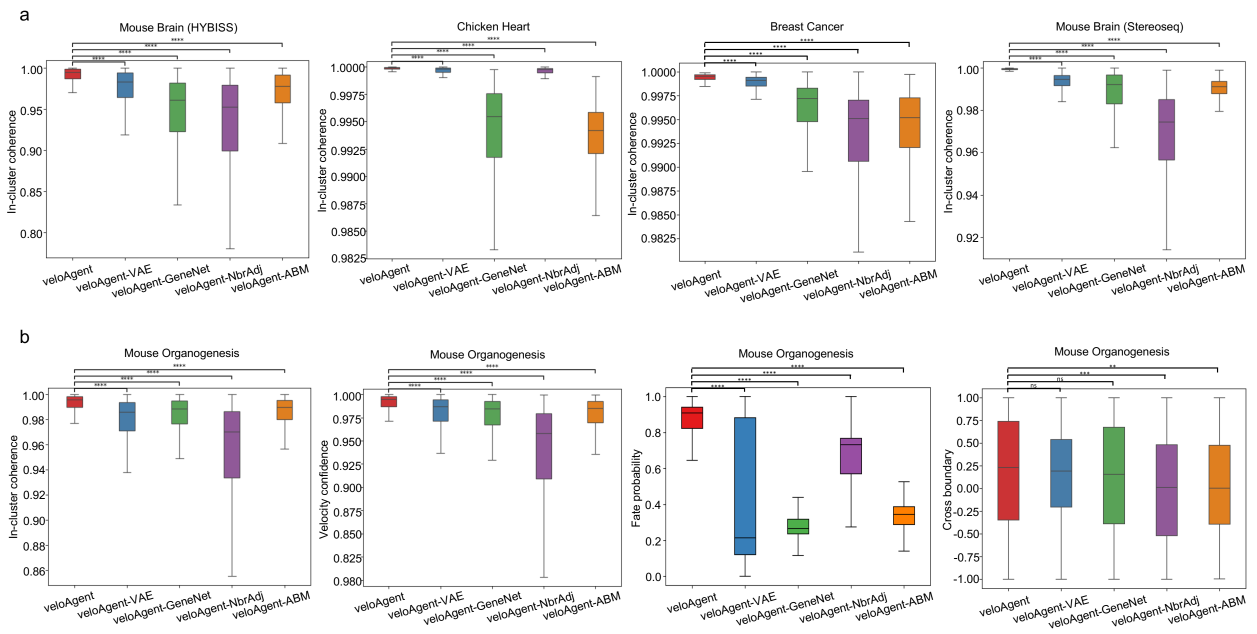

Appendix Figure S14: Extended ablation results for spatial transcriptomics datasets. a, In-cluster coherence scores across all ablation models for four spatial transcriptomics datasets (left to right): mouse brain (HybISS), chicken heart, breast cancer, and mouse brain (Stereo-seq). **b,** In-cluster coherence (left), velocity confidence (middle left), fate probability (middle right) and cross boundary (right) scores across ablation models for the seq-FISH mouse organogenesis dataset. Performance drops in ablated models demonstrate that the full model consistently outperforms alternatives and that each component is necessary for accurate RNA velocity inference. Error bars show interquartile range. Statistical significance calculated using Mann-Whitney U-test with FDR correction. Significance levels are indicated as follows: n.s, not significant ($P \geq 0.05$); * ($P < 0.05$); ** ($P < 0.01$); *** ($P < 0.001$); and **** ($P < 0.0001$).

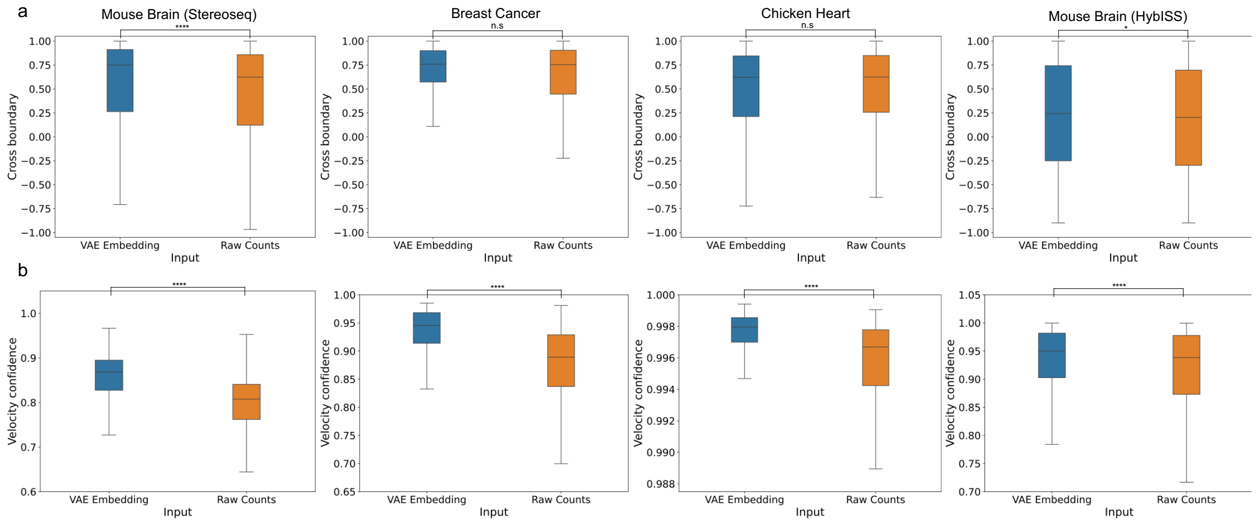

Appendix Figure S15: Quantitative performance of veloAgent with and without using VAE. a, Cross-boundary scores of veloAgent using the VAE embedding as input to the gene-gene neural network, compared with using raw unspliced and spliced counts as input. This metric measures the correctness of predicted transitions across annotated cluster boundaries based on ground-truth developmental labels. **b,** Velocity confidence scores of veloAgent using the VAE embedding as input to the gene-gene neural network, compared to using raw unspliced and spliced counts as input. This metric quantifies how well predicted velocities align with local gene expression structure. Higher scores indicate more reliable predictions. Error bars show interquartile range. Statistical significance calculated using Mann-Whitney U-test with FDR correction. Significance levels are indicated as follows: n.s, not significant ($P \geq 0.05$); * ($P < 0.05$); ** ($P < 0.01$); *** ($P < 0.001$); and **** ($P < 0.0001$).

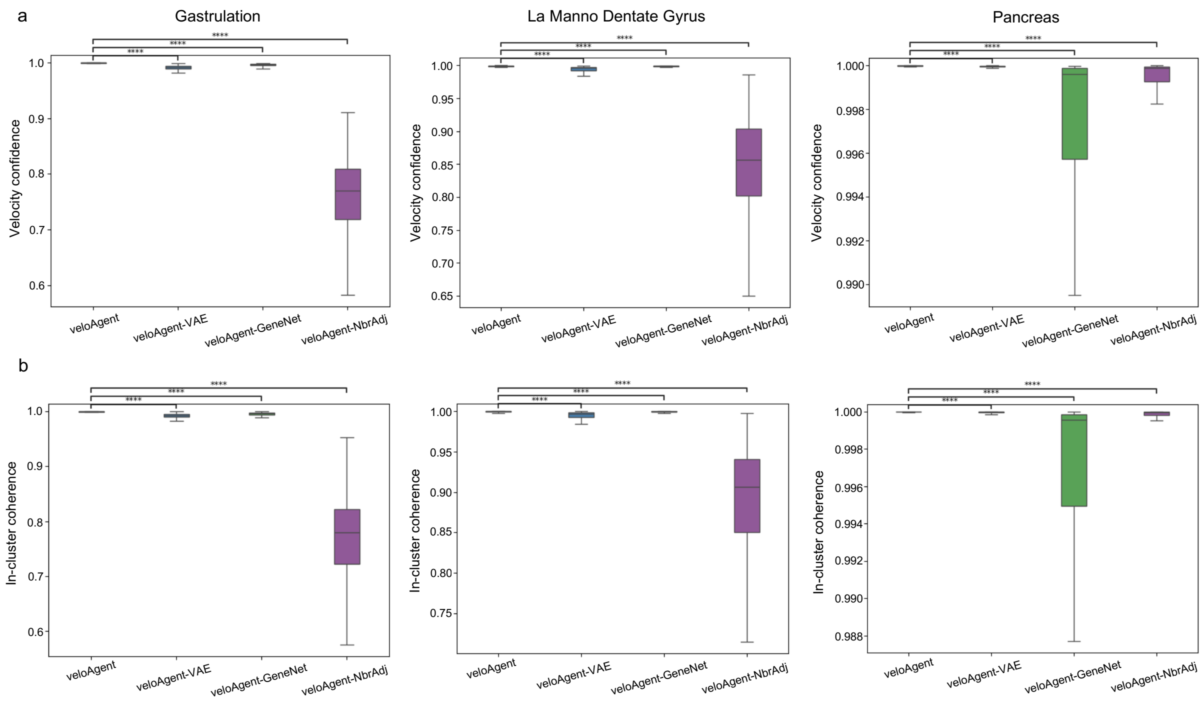

Appendix Figure S16: Ablation of veloAgent's components on non-spatial datasets. Evaluation of the contribution of individual model components: variational autoencoder (VAE), gene-gene network, and neighbor adjustment; removing one component at a time. **a,** Velocity confidence scores across all ablation models for three non-spatial datasets: gastrulation (left), dentate gyrus (middle), and pancreas (right). **b,** In-cluster coherence scores across ablation models. Performance drops observed in both metrics demonstrate that each component is necessary and contributes to the overall effectiveness of veloAgent. Error bars show interquartile range. Statistical significance calculated using Mann-Whitney U test with FDR correction. Significance levels are indicated as follows: n.s, not significant ($P \geq 0.05$); * ($P < 0.05$); ** ($P < 0.01$); *** ($P < 0.001$); and **** ($P < 0.0001$).

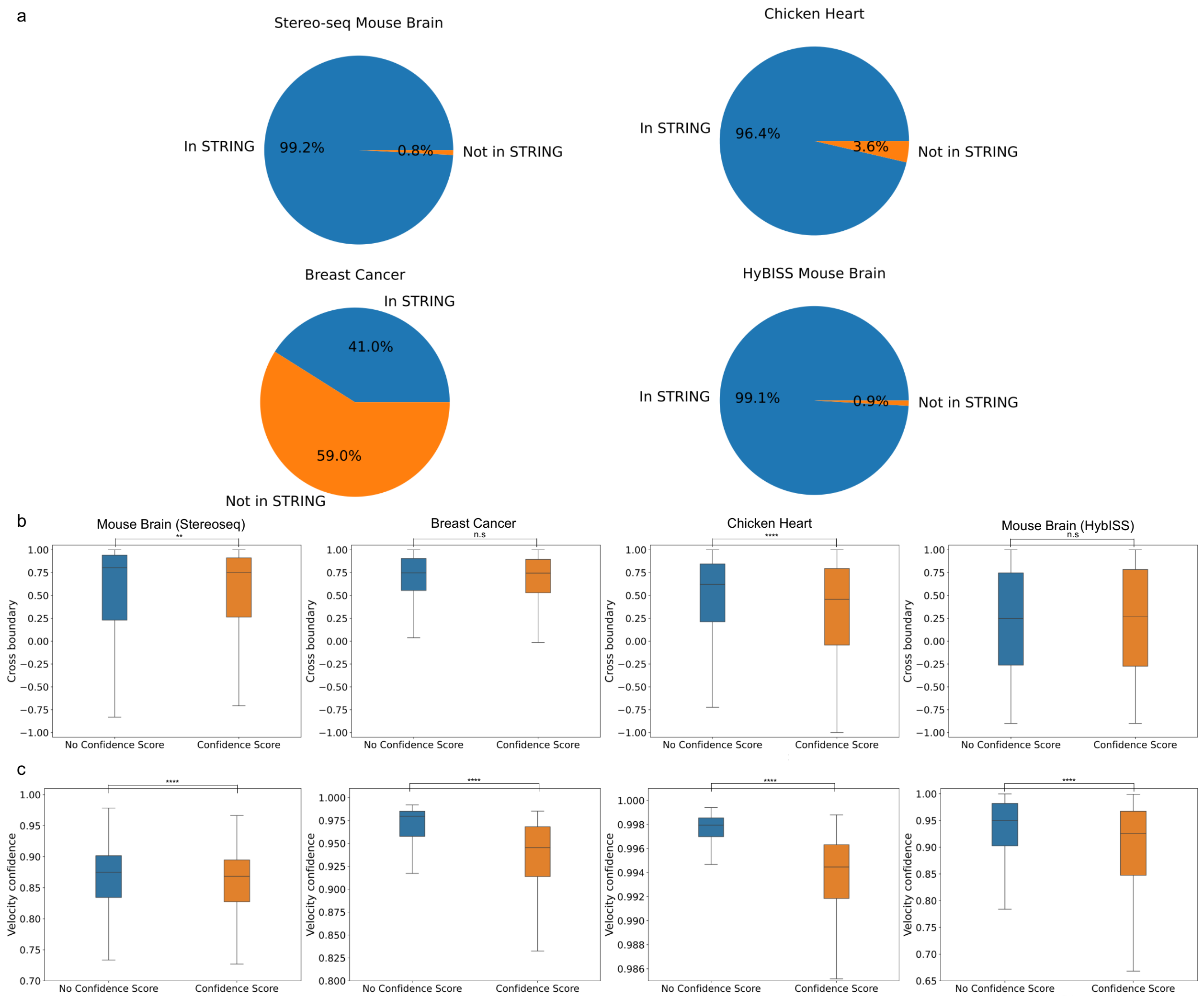

Appendix Figure S17: Evaluation of incorporating gene-gene interaction prior from STRING. a, Pie charts show the proportion of genes in each dataset that are present in STRING and therefore incorporated as prior knowledge in the gene-gene neural network. **b,** Cross-boundary scores of veloAgent with and without applying confidence score filtering (combined score greater than 400; medium confidence or better) to restrict gene-gene interactions in the neural network. This metric measures the correctness of predicted transitions across annotated cluster boundaries based on ground-truth developmental labels. Higher scores reflect better agreement with known trajectories. **c,** Velocity confidence scores of veloAgent with and without applying confidence score filtering (combined score greater than 400; medium confidence or better) to restrict STRING-derived gene-gene interactions in the neural network. This metric quantifies how well

predicted velocities align with local gene expression structure. Higher scores indicate more reliable predictions. Error bars show interquartile range. Statistical significance calculated using Mann-Whitney U test with FDR correction. Significance levels are indicated as follows: n.s, not significant ($P \geq 0.05$); * ($P < 0.05$); ** ($P < 0.01$); *** ($P < 0.001$); and **** ($P < 0.0001$).

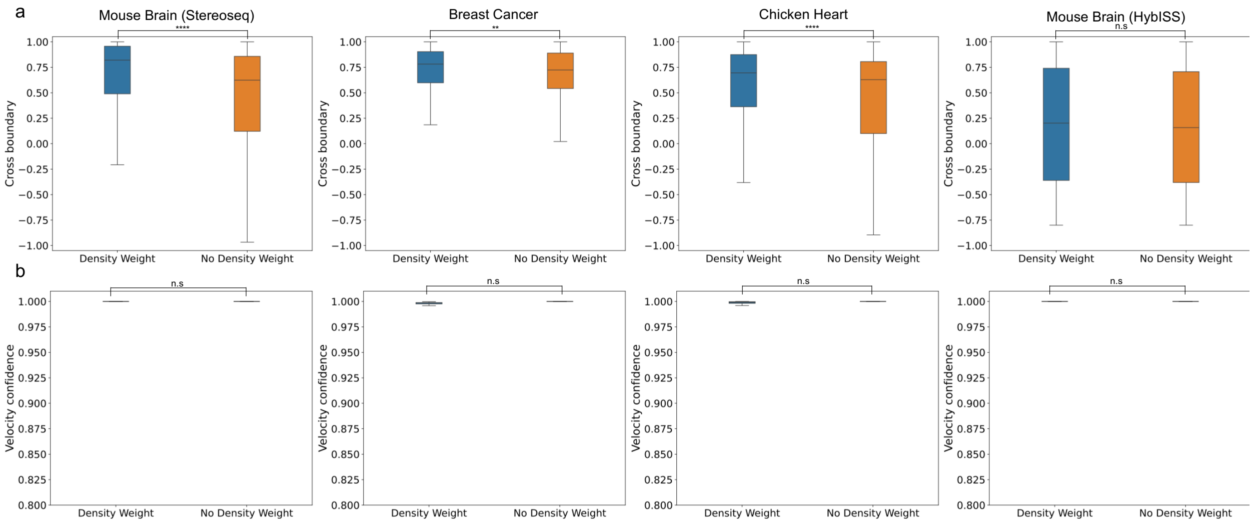

Appendix Figure S18: Quantitative performance of veloAgent with and without the density weight (DW) in the ABM. a, Cross-boundary scores of veloAgent with and without the density weight (DW) in the ABM. This metric measures the correctness of predicted transitions across annotated cluster boundaries based on ground-truth developmental labels. Higher scores reflect better agreement with known trajectories. **b,** Velocity confidence scores of veloAgent with and without the density weight (DW). This metric quantifies how well predicted velocities align with local gene expression structure. Higher scores indicate more reliable predictions. Error bars show interquartile range. Statistical significance calculated using Mann-Whitney U test with FDR correction. Significance levels are indicated as follows: n.s, not significant ($P \geq 0.05$); * ($P < 0.05$); ** ($P < 0.01$); *** ($P < 0.001$); and **** ($P < 0.0001$).

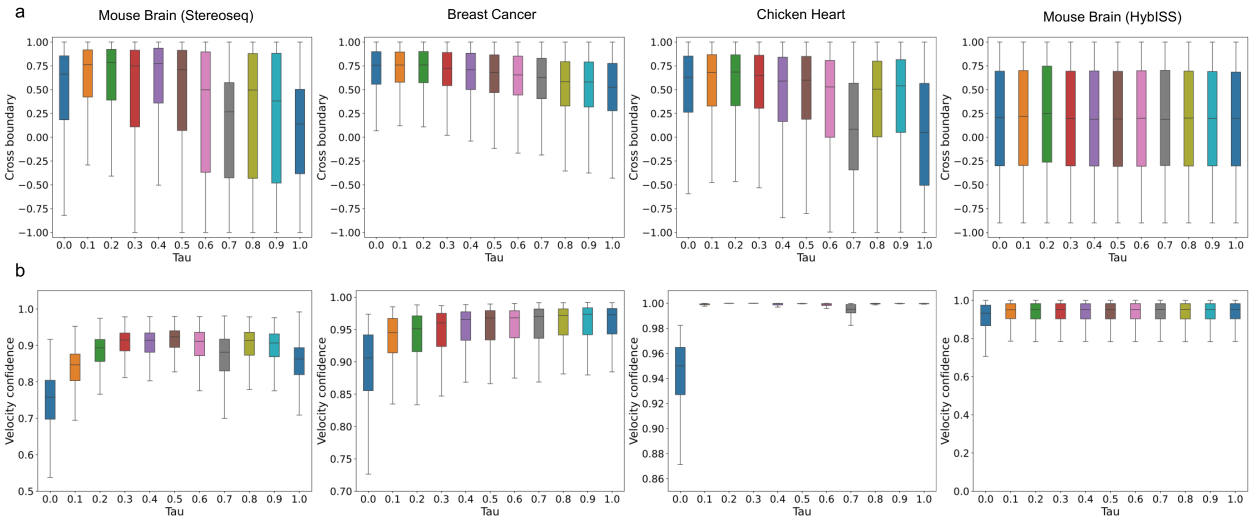

Appendix Figure S19: Quantitative performance of veloAgent using different τ values. a, Cross-boundary scores across τ values ranging from 0-1 (inclusive). This metric measures the correctness of predicted transitions across annotated cluster boundaries, based on ground-truth developmental labels. Higher scores reflect better agreement with known trajectories. **b,** Velocity confidence scores across τ values ranging from 0-1 (inclusive). This metric quantifies how well predicted velocities align with local gene expression structure. Higher scores indicate more reliable predictions. $\tau = 0.2$ is the default value used in veloAgent. Error bars show interquartile range.

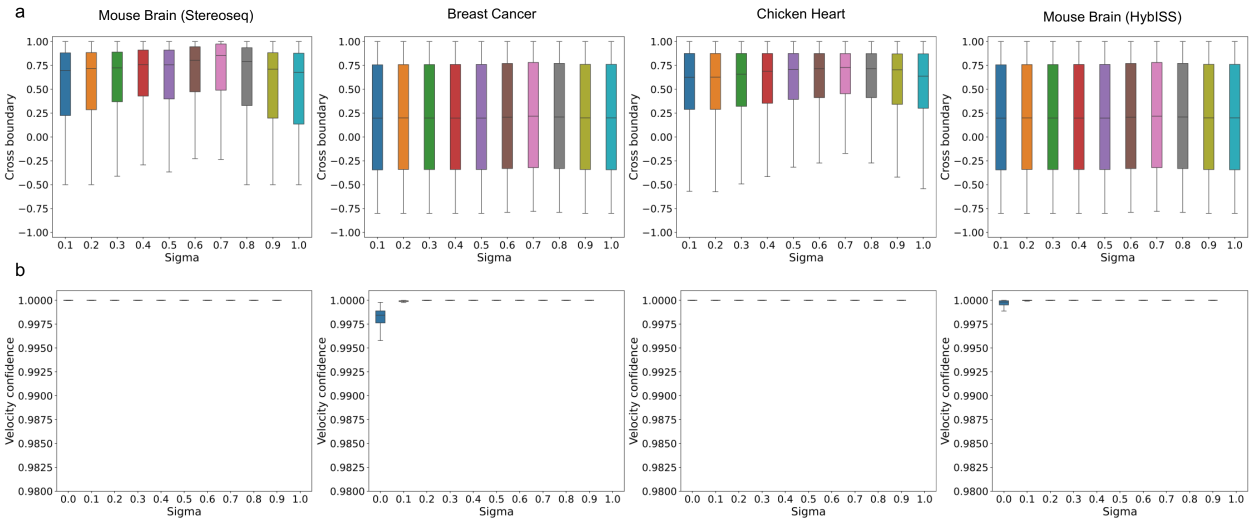

Appendix Figure S20: Quantitative performance of veloAgent across different σ_{ABM} values. **a,** Cross-boundary scores for σ_{ABM} values ranging from 0-1 (inclusive). This metric measures the correctness of predicted transitions across annotated cluster boundaries based on ground-truth developmental labels. Higher scores reflect better agreement with known trajectories. **b,** Velocity confidence scores for σ_{ABM} values ranging from 0-1 (inclusive). This metric quantifies how well predicted velocities align with local gene expression structure. Higher scores indicate more reliable predictions. $\sigma_{ABM} = 0.7$ is the default value used in veloAgent. Error bars show interquartile range.

Appendix Supplementary Tables

Method	Cross Boundary	Fate Probability	Velocity Confidence
**Stereo-seq**			
cellDancer	1.821E-111	< 1E-308	6.344E-05
SIRV	3.014E-161	< 1E-308	< 1E-308
DeepVelo	7.241E-84	< 1E-308	< 1E-308
veloVAE	9.548E-107	< 1E-308	< 1E-308
veloVI	6.984E-01	6.990E-132	< 1E-308
UniTVelo	1.500E-70	< 1E-308	< 1E-308
scVelo	2.831E-68	< 1E-308	< 1E-308
**Breast Cancer**			
cellDancer	3.588E-60	< 1E-308	< 1E-308
SIRV	2.237E-150	< 1E-308	< 1E-308
DeepVelo	2.647E-113	< 1E-308	< 1E-308
veloVAE	1.106E-171	< 1E-308	< 1E-308
veloVI	9.286E-103	< 1E-308	< 1E-308
UniTVelo	4.803E-79	< 1E-308	< 1E-308
scVelo	7.247E-69	< 1E-308	< 1E-308
**Chicken Heart**			
cellDancer	2.923E-03	< 1E-308	< 1E-308
SIRV	5.450E-27	< 1E-308	< 1E-308
DeepVelo	1.220E-26	4.187E-19	< 1E-308
veloVAE	5.709E-106	< 1E-308	< 1E-308
veloVI	1.032E-35	< 1E-308	< 1E-308
UniTVelo	3.859E-48	< 1E-308	< 1E-308
scVelo	8.872E-37	< 1E-308	< 1E-308
**HyBISS**			
cellDancer	5.976E-07	1.071E-51	< 1E-308
SIRV	3.880E-03	3.298E-303	< 1E-308
DeepVelo	5.411E-04	< 1E-308	< 1E-308
veloVAE	3.912E-15	7.556E-150	< 1E-308
veloVI	8.912E-05	< 1E-308	< 1E-308
UniTVelo	9.538E-03	< 1E-308	< 1E-308
scVelo	8.400E-03	< 1E-308	< 1E-308

Appendix Table S1. *P*-values for each metric across spatial transcriptomics datasets for veloAgent and each benchmarked method.

Method	Velocity Confidence	Cross Boundary	In-cluster Coherence
**Gastrulation**			
cellDancer	< 1E-308	< 1E-308	< 1E-308
DeepVelo	< 1E-308	5.444E-25	< 1E-308
veloVAE	< 1E-308	8.671E-25	< 1E-308
veloVI	< 1E-308	4.177E-06	< 1E-308
UniTVelo	< 1E-308	< 1E-308	9.783E-01
scVelo	< 1E-308	2.976E-81	< 1E-308
**Dentate Gyrus**			
cellDancer	< 1E-308	2.217E-222	< 1E-308
DeepVelo	< 1E-308	9.509E-01	< 1E-308
veloVAE	< 1E-308	9.978E-01	< 1E-308
veloVI	< 1E-308	3.132E-46	< 1E-308
UniTVelo	< 1E-308	9.306E-37	< 1E-308
scVelo	< 1E-308	4.083E-51	< 1E-308
**Pancreas**			
cellDancer	< 1E-308	8.319E-11	< 1E-308
DeepVelo	< 1E-308	9.371E-01	< 1E-308
veloVAE	< 1E-308	6.104E-05	< 1E-308
veloVI	< 1E-308	9.509E-01	< 1E-308
UniTVelo	< 1E-308	9.416E-01	< 1E-308
scVelo	< 1E-308	2.547E-10	< 1E-308

Appendix Table S2. *P*-values for each metric across scRNA-seq datasets for veloAgent and each benchmarked method.

Method	Cross Boundary	Spatial Velocity Conf.	Spatial Fate Probability
**Stereo-seq**			
cellDancer	1.051E-27	5.867E-248	< 1E-308
SIRV	9.316E-19	4.406E-248	< 1E-308
DeepVelo	1.733E-01	4.406E-248	< 1E-308
veloVAE	1.658E-03	4.406E-248	< 1E-308
veloVI	7.360E-01	4.406E-248	< 1E-308
UniTVelo	6.845E-35	4.406E-248	< 1E-308
scVelo	1.388E-20	4.406E-248	< 1E-308
**Breast Cancer**			
cellDancer	2.885E-13	2.576E-74	1.168E-10
SIRV	5.314E-33	1.969E-79	1.351E-02
DeepVelo	3.192E-02	1.969E-79	5.308E-186
veloVAE	3.606E-30	1.969E-79	1.262E-65
veloVI	3.412E-31	1.969E-79	2.269E-06
UniTVelo	1.490E-09	1.968E-79	1.451E-05
scVelo	2.646E-05	2.146E-81	8.469E-40
**Chicken Heart**			
cellDancer	3.453E-19	5.397E-51	8.071E-101
SIRV	4.325E-02	5.397E-51	< 1E-308
DeepVelo	3.631E-02	5.397E-51	< 1E-308
veloVAE	4.911E-20	5.397E-51	1.119E-307
veloVI	2.462E-11	5.397E-51	< 1E-308
UniTVelo	5.793E-09	5.397E-51	< 1E-308
scVelo	8.873E-16	5.397E-51	< 1E-308
**HyBISS**			
cellDancer	2.041E-02	3.157E-105	5.249E-40
SIRV	2.041E-02	3.157E-105	1.423E-275
DeepVelo	2.041E-02	3.157E-105	< 1E-308
veloVAE	2.041E-02	3.157E-105	< 1E-308
veloVI	2.041E-02	3.157E-105	< 1E-308
UniTVelo	3.592E-09	3.157E-105	< 1E-308
scVelo	2.041E-02	3.157E-105	< 1E-308

Appendix Table S3. *P*-values for each spatial metric across spatial transcriptomics datasets for veloAgent and each benchmarked method.

Method	Cross Boundary	Spatial Fate Probability	Spatial Velocity Conf.
**Stereo-seq**			
VAE	5.57E-09	3.98E-02	< 1E-308
GG-Net	2.01E-171	< 1E-308	< 1E-308
Nbr-Adj	8.49E-08	< 1E-308	< 1E-308
ABM	9.86E-28	< 1E-308	< 1E-308
**Breast Cancer**			
VAE	3.04E-21	< 1E-308	< 1E-308
GG-Net	4.956-81	< 1E-308	< 1E-308
Nbr-Adj	2.61E-61	< 1E-308	< 1E-308
ABM	3.87E-60	< 1E-308	< 1E-308
**Chicken Heart**			
VAE	4.63E-02	< 1E-308	8.14E-24
GG-Net	5.04E-184	< 1E-308	< 1E-308
Nbr-Adj	3.13E-02	4.00E-02	3.34E-26
ABM	3.31E-85	< 1E-308	< 1E-308
**HyBISS**			
VAE	5.51E-04	< 1E-308	5.37E-120
GG-Net	1.25E-05	< 1E-308	< 1E-308
Nbr-Adj	9.02E-06	< 1E-308	< 1E-308
ABM	3.91E-05	< 1E-308	2.22E-112

Appendix Table S4. *P*-values for each spatial metric across spatial transcriptomics datasets for veloAgent and ablated versions of the model.
