# Supplementary material for: Dissecting and steering cell dynamics using spatially-informed RNA velocity with veloAgent
Source: Mol Syst Biol. 2026 May 6;22(7):1180–200. doi: 10.1038/s44320-026-00213-w (PMC13328566; doi:10.1038/s44320-026-00213-w)
Supplement: Supplementary file 1 — Peer Review File [file 44320_2026_213_MOESM1_ESM.pdf]

molecular systems biology

Dissecting and steering cell dynamics using spatially-informed RNA velocity with veloAgent

Vishvak Raghavan, Brent Yoon, Gregory Fonseca, Yue Li, and Jun Ding

Corresponding author(s): Jun Ding (jun.ding@mcgill.ca)

Review Timeline:

Submission Date:	8th Jan 26
Editorial Decision:	12th Feb 26
Revision Received:	20th Mar 26
Editorial Decision:	2nd Apr 26
Revision Received:	9th Apr 26
Accepted:	16th Apr 26

Editor: Jingyi Hou

Transaction Report:

(Note: With the exception of the correction of typographical or spelling errors that could be a source of ambiguity, letters and reports are not edited. Depending on transfer agreements, referee reports obtained elsewhere may or may not be included in this compilation. Referee reports are anonymous unless the Referee chooses to sign their reports.)

12th Feb 2026

Manuscript Number: MSB-2026-13571-T
Title: Dissecting and steering cell dynamics using spatially-informed RNA velocity with veloAgent
Author: Jun Ding
Brent Yoon
Vishvak Raghavan

Dear Dr. Ding,

Thank you for submitting your work to Molecular Systems Biology. We have now heard back from the three reviewers who agreed to evaluate your manuscript. As you will see from the comments below that they find the study interesting and relevant. They raise, however, several important points, which should be convincingly addressed in a revision of this work.

I think that the recommendations of the reviewers are rather straightforward so there is no need to repeat the points listed below. All issues raised by the reviewers need to be satisfactorily addressed. As you may already know, our editorial policy allows in principle a single round of major revision so it is essential to provide responses to the reviewers' comments that are as complete as possible. Please feel free to contact me in case you would like to discuss in further detail any of the issues raised by the reviewers.

On a more editorial level, we would ask you to address the following issues:

- Please provide a .docx formatted version of the manuscript text (including legends for main figures, EV figures and tables). Please make sure that the changes are highlighted to be clearly visible.

- Please provide individual production quality figure files as .eps, .tif, .jpg (one file per figure). When assembling figures, please refer to our figure preparation guideline in order to ensure proper formatting and readability in print as well as on screen: https://media.springernature.com/original/springer-cms/rest/v1/content/27825798/data/v1

-Please provide a .docx formatted letter INCLUDING the reviewers' reports and your detailed point-by-point responses to their comments. As part of the EMBO Press transparent editorial process, the point-by-point response is part of the Review Process File (RPF), which will be published alongside your paper.

-Please note that all corresponding authors are required to supply an ORCID ID for their name upon submission of a revised manuscript.

-We replaced Supplementary Information with Expanded View (EV) Figures and Tables that are collapsible/expandable online. A maximum of 5 EV Figures can be typeset. EV Figures should be cited as 'Figure EV1, Figure EV2" etc... in the text and their respective legends should be included in the main text after the legends of regular figures.

Additional Tables/Datasets should be labeled and referred to as Table EV1, Dataset EV1, etc. Legends have to be provided in a separate tab in case of .xls files. Alternatively, the legend can be supplied as a separate text file (README) and zipped together with the Table/Dataset file.

For the figures and tables that you do NOT wish to display as Expanded View figures, they should be bundled together with their legends in a single PDF file called *Appendix*, which should start with a short Table of Content. Each legend should be below the corresponding Figure/Table in the Appendix. Appendix figures and tables should be referred to in the main text as: "Appendix Figure S1, Appendix Figure S2, Appendix Table S1" etc. See detailed instructions regarding expanded view here: https://link.springer.com/journal/44320/submission-guidelines#cms-Expanded-View-data.

-Before submitting your revision, primary datasets (and computer code, where appropriate) produced in this study need to be deposited in an appropriate public database.
Please remember to provide a reviewer password if the datasets are not yet public.
The accession numbers and database should be listed in a formal "Data Availability" section (placed after Materials & Method) that follows the model below (see also https://link.springer.com/partners/embo-press/editorial-policies#Data%20availability%20statement). Please note that the Data Availability Section is restricted to new primary data that are part of this study.
Data availability
The datasets (and computer code) produced in this study are available in the following databases:
- RNA-Seq data: Gene Expression Omnibus GSE46843 (https://www.ncbi.nlm.nih.gov/geo/query/acc.cgi?acc=GSE46843)
- [data type]: [name of the resource] [accession number/identifier/doi] ([URL or identifiers.org/DATABASE:ACCESSION])

*** Note - All links should resolve to a page where the data can be accessed. ***

- At EMBO Press we ask authors to provide source data for the main manuscript figures. You will receive a separate email with instructions for providing source data with your revised manuscript, including how to upload and organize the files.
Additional information on source data and instruction on how to label the files are available < https://link.springer.com/journal/44320/submission-guidelines#cms-Source-data >

- Our journal encourages inclusion of *data citations in the reference list* to directly cite datasets that were re-used and obtained from public databases. Data citations in the article text are distinct from normal bibliographical citations and should directly link to the database records from which the data can be accessed. In the main text, data citations are formatted as follows: "Data ref: Smith et al, 2001". In the Reference list, data citations must be labeled with "[DATASET]". A data reference must provide the database name, accession number/identifiers and a resolvable link to the landing page from which the data can be accessed at the end of the reference. Further instructions are available at < https://link.springer.com/journal/44320/submission-guidelines#cms-Reference-guidelines >.

- We updated our journal's competing interests policy in January 2022 and request authors to consider both actual and perceived competing interests. Please review the policy (https://link.springer.com/partners/embo-press/editorial-policies#Competing%20interest%20disclosures) and update your competing interests if necessary.
Please use the heading "Disclosure statement and competing interests".

- All Materials and Methods need to be described in the main text using our 'Structured Methods' format. According to this format, the Methods section includes a Reagents and Tools Table (listing key reagents, experimental models, software and relevant equipment and including their sources and relevant identifiers) followed by a Methods and Protocols section describing the methods, ideally using a step-by-step protocol format. The aim is to facilitate adoption of the methodologies across labs.

Please download and fill our Reagents and Tools Table template (.docx), which you can find in our author guidelines: https://link.springer.com/journal/44320/submission-guidelines#structuredmethods.

When submitting your revised manuscript, please do not include the Reagents and Tools Table in the Methods section of the manuscript but upload it as a separate file choosing the file type "Reagent Table".

-Regarding data quantification:
Please ensure to specify the name of the statistical test used to generate error bars and P values, the number (n) of independent experiments (please specify technical or biological replicates) underlying each data point and the test used to calculate p-values in each figure legend. Discussion of statistical methodology can be reported in the materials and methods section, but figure legends should contain a basic description of n, P and the test applied.
Graphs must include a description of the bars and the error bars (s.d., s.e.m.).
Please also include scale bars in all microscopy images.

- Please provide a "standfirst text" summarizing the study in one or two sentences (approximately 250 characters, including space), three to four "bullet points" highlighting the main findings and a " visual abstract" (550px width and 400-600 px height, PNG format) to highlight the paper on our homepage.
Please refer to published papers for examples.

When you resubmit your manuscript, please download our CHECKLIST (https://media.springernature.com/original/springer-cms/rest/v1/content/27825796/data/v1) and include the completed form in your submission.
Please note that the Author Checklist will be published alongside the paper as part of the transparent process .

If you feel you can satisfactorily deal with these points and those listed by the referees, you may wish to submit a revised version of your manuscript. Please attach a covering letter giving details of the way in which you have handled each of the points raised by the referees. A revised manuscript will be once again subject to review and you probably understand that we can give you no guarantee at this stage that the eventual outcome will be favorable.

I look forward to receiving the revised manuscript soon.

Kind regards,
Jingyi

Jingyi Hou
Senior Editor
Molecular Systems Biology

*** PLEASE NOTE *** As part of the EMBO Press transparent editorial process initiative (see our Editorial at https://dx.doi.org/10.1038/msb.2010.72), Molecular Systems Biology publishes online a Review Process File with each accepted manuscripts. This file will be published in conjunction with your paper and will include the anonymous referee reports, your point-by-point response and all pertinent correspondence relating to the manuscript. If you do NOT want this File to be published, please inform the editorial office at contact@molsystbiol.org within 14 days upon receipt of the present letter.

Reviewer #1:

Summary
This paper presents veloAgent, a promising framework that integrates deep learning with spatial context to estimate RNA velocity.

Major Comments

1. It would be helpful to see more detail on the extraction of spliced and unspliced counts, particularly given that Visium's poly-A capture efficiency for introns may be lower than scRNA-seq. I suggest clarifying the specific pipeline used and discussing how potential sparsity in unspliced reads might influence the model's reliability.

2. To better quantify the specific benefit of the spatial information, I recommend including an ablation study comparing veloAgent with and without the spatial module enabled. This would help clarify whether performance gains are driven by the spatial context or the underlying neural network architecture.

3. Applying kinetic equations designed for single cells directly to multi-cell Visium spots may result in averaged dynamics that obscure true cell states.

4. Regarding the statement on Line 155 about qualitatively superior performance, it would be helpful to cite the specific figure panels that demonstrate this. Please specify the visual criteria or improvements the reader should focus on to support this claim.

5. Since current benchmarks primarily evaluate gene expression similarity, it would be valuable to incorporate metrics that specifically assess spatial accuracy. Using spatially-aware metrics (whether existing or newly proposed) would strengthen the evaluation of the model's spatial performance.

6. Visualizing velocity arrows directly on the spatial map can be potentially misleading, as it may imply physical migration rather than differentiation state changes.

Reviewer #2:

RNA velocity inference has garnered significant attention, prompting the development of numerous computational frameworks. However, this study presents an innovative deep learning framework veloAgent that addresses several key limitations in current velocity analyses. veloAgent is one of the first methods that can infer spatially resolved RNA velocity through deep integration of spatial transcriptomics data into the velocity inference process via an Agent-Based Model (ABM). This integration enbles the inference of cell trajectories that are anatomically more plausible and the influence of the tissue microenvironment. To overcome the scalability bottlenecks of existing methods, veloAgent adopts a gene-centric model architecture whose computational complexity does not increase drastically with the number of cells. More importantly and interestingly, this study introduces the field's first in silico perturbation module, which transforms RNA velocity from a descriptive tool into a causal inference system capable of identifying key regulatory factors. By incorporating prior knowledge of gene interactions into neural network, veloAgent also improves the model's biological interpretability. The authors demonstrate the superior performance of veloAgent on several datasets with varying technologies and through a solid comparison analysis with other methods.

Overall, this new approach represents a significant advance over existing methods, and serves as a unique and useful tool for inferring spatially resolved RNA velocity and manipulating spatial velocity to simulate regulatory interventions. However, I still have a few comments, especially on the method details, that should be addressed.

1. It is important to performance the sensitive analysis of the regularization parameters lambda and tau in the overall loss function. High parameter values will likely lead to over-smoothed RNA velocity across cell neighbors. A potential problem may also exist in the ABM-based model of spatial velocity. While this is challenging problem in the field, is there any suggestion to balance the similarity and sharpness of a cell's RNA velocity against its neighbors?

2. In the ABM part, please clarify the meaning of the variable in equation 10 and how to compute it. In equation 12, Is DV_i the

velocity calculated after Phase 3? Should DW_i be also added in the second term related to the cell neighbors? How was the performance if the density weight was not included in equation 12?

3. Using prior gene interaction information appears to reduce computational complexity, but the fully-connected approach for genes without prior information weakens this advantage. Can the authors clarify the proportion of genes without prior information? In addition, how many genes are used in this Phase? What is the threshold of confidence scores when selecting gene-gene interactions from STRING database?

4. Method details of hyperparameter settings are missing. For example, the learning rate, optimizer choice, batch size, and the latent dimension size of the VAE, the radius r, the sigma and the lambda in the ABM method. Please check all hyperparameters and clarify the default settings.

5. What is the meaning of the colors in the gene velocity plots such as Fig. 2e and Fig. 3f? The font size of GO terms in several figure panels are very small. It would be helpful to increase it.

Reviewer #3:

The manuscript presents veloAgent, a hybrid deep learning and agent-based framework for estimating RNA velocity by integrating gene expression data with prior knowledge of gene-gene interactions and spatial context. The method involves several stages: a variational autoencoder encodes spliced and unspliced mRNA counts into a latent space; a constrained neural network infers cell- and gene-specific kinetic parameters; and an agent-based module refines velocity vectors using transcriptional similarity, local density, and intercellular distance. The authors also demonstrate the use of in silico perturbations to assess gene-level contributions to trajectory directionality and highlight the model's scalability across large and multi-batch datasets. Notably, the authors perform ablation studies on individual components of the model-a practice that is rare in similar studies.

However, I would like to raise the following questions regarding certain methodological choices, implementation details, and biological interpretations.

1. (Line 612) The description of "sparse connections informed by gene-gene interaction data" lacks implementation details. Please clarify how the interaction data are incorporated into the model architecture.
2. (Line 616) Is it reasonable to connect genes without known interactions to all other gene nodes? This design seems biologically implausible and may introduce noise.
3. (Line 634) The loss function appears similar to that of CellDancer, but no citation is provided. Please acknowledge prior work if applicable.
4. (Line 634) CellDancer uses raw spliced/unspliced counts as input, whereas this work uses VAE-derived embeddings. Given potential information loss from VAE compression, does this design offer advantages over CellDancer? Note that CellDancer could also be extended with gene-gene interaction priors.
5. (Line 651) How were the hyperparameters λ and τ in loss function (8) chosen? Their selection is critical given the multi-component loss.
6. (Lines 666-689) Several variables are not clearly defined, e.g., ρ in Equation (10). Please specify their definition.
7. (Lines 666-689) The term "agent-based module" may be misleading, as the module only performs post-hoc velocity refinement and does not involve autonomous agents. Consider using a more descriptive name.
8. (Lines 666-689) How should the hyperparameters σ_ABM, λ_ABM, and neighborhood radius r be selected? In particular, how is r determined for different spatial transcriptomics technologies?
9. (Lines 691-696) The in silico perturbation procedure lacks detail. Please clarify: which parameters (e.g., transcription rate α) are modified, and which parts of the model remain active during re-computation (without retraining).

Dear Dr. Hou,
We would like to express our gratitude to the reviewers and editorial team for their constructive comments and suggestions offered on our manuscript, titled "Dissecting and steering cell dynamics using spatially-informed RNA velocity with veloAgent" with manuscript ID MSB-2026-13571-T. The detailed feedback has substantially improved the manuscript's scientific rigor and clarity. We also thank the editor for the expedient and careful handling of our submission. Following the guidance provided, we have addressed each comment in detail. Enclosed are our point-by-point responses to the issues raised and the amendments applied to the manuscript (all changes were tracked in blue).

Reviewer 1

General Comments: This paper presents veloAgent, a promising framework that integrates deep learning with spatial context to estimate RNA velocity.

Response: Thank you for your encouraging comments and for recognizing the potential of veloAgent. To further improve clarity, we have refined the manuscript and expanded on certain design decisions underlying our approach. We appreciate the thoughtful suggestions that have helped improve the manuscript.

Comment 1: It would be helpful to see more detail on the extraction of spliced and unspliced counts, particularly given that Visium's poly-A capture efficiency for introns may be lower than scRNA-seq. I suggest clarifying the specific pipeline used and discussing how potential sparsity in unspliced reads might influence the model's reliability.

Response: Thank you for pointing out this important observation. We have revised the preprocessing section to clarify that spliced and unspliced count matrices were obtained using the standard velocyto pipeline. For datasets where these matrices were not already available, we generated them ourselves using the standard velocyto workflow; for datasets with publicly available processed matrices, we used the provided spliced and unspliced counts directly. We now explicitly state in the Methods that this preprocessing follows the standard velocyto procedure, using its default settings unless otherwise specified.

We also added a discussion noting that Visium's poly-A capture chemistry may yield lower intronic capture efficiency relative to scRNA-seq, potentially increasing sparsity in unspliced reads. We clarify that veloAgent partially alleviates this limitation through a combination of latent embedding and the agent-based modeling (ABM) refinement module. The latent representation provides a denoised encoding of transcriptional states, while the ABM module aggregates information from neighboring cells based on spatial proximity and transcriptional similarity, helping to stabilize velocity estimation in sparse settings. This effect is further supported by our

ablation analyses (Figure 6c–f). This discussion has been added in the "Single-cell sequencing data preprocessing" section of the Methods. Furthermore, we would like to note that our evaluation was not limited to Visium datasets. We also included spatial transcriptomics datasets generated using higher-resolution platforms, such as Stereo-seq, which provide near single-cell resolution and improved spatial granularity. The consistent performance observed across these technologies further supports the robustness of veloAgent to differences in spatial resolution and intronic capture efficiency.

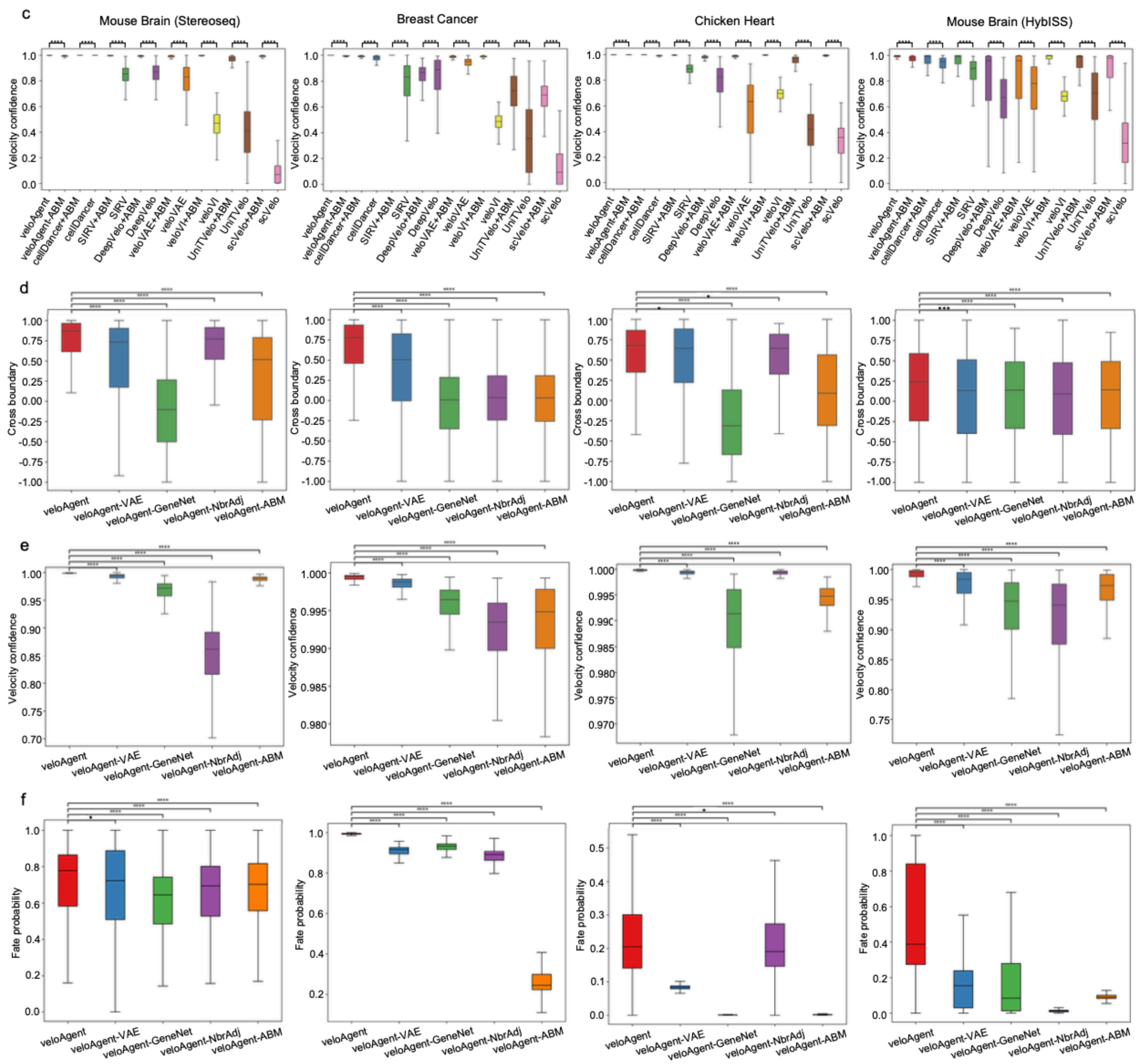

Figure 6 Ablation study of veloAgent. **c,** Comparative evaluation of velocity confidence scores before and after applying veloAgent's agent-based model (ABM) for spatial refinement on top of each method's temporal velocity estimates. Original estimates from each method are contrasted with their spatially adjusted counterparts, showing consistent improvement when spatial data is incorporated. **d-f,** To assess the contribution of key model components, an ablation study was performed by systematically removing

each module from veloAgent, including the ABM module. **d,** Cross-boundary accuracy, **e,** velocity confidence and **f,** fate probability metrics across four configurations: full veloAgent, without latent-space smoothing, without gene-informed network architecture, without neighbour adjustment and without incorporating spatial transcriptomics data using agent-based modeling. Performance drops observed in ablated models validate the importance of each component. Error bars show interquartile range. Statistical significance calculated using Mann-Whitney U-test with FDR correction. Significance levels are indicated as follows: n.s, not significant ($P \geq 0.05$); * ($P<0.05$); ** ($P<0.01$); *** ($P<0.001$); and **** ($P<0.0001$). Exact P-values can be found in Appendix Table S4.

Comment 2: To better quantify the specific benefit of the spatial information, I recommend including an ablation study comparing veloAgent with and without the spatial module enabled. This would help clarify whether performance gains are driven by the spatial context or the underlying neural network architecture.

Response: Thank you for this critical ablation comment. We agree that isolating the contribution of spatial information is important for interpreting performance gains. We would like to clarify that we have now performed ablation analyses comparing veloAgent with and without the spatial ABM module.

Specifically, our ablation experiments (Fig. 6d-f) systematically remove each major component of the framework, including the ABM spatial refinement module. Across multiple complementary metrics (cross-boundary direction, fate probability, velocity confidence), the full model consistently outperforms the corresponding ablated variants. Notably, removal of the ABM module leads to the largest performance decrease among all components, indicating that spatial information is a major contributor to the observed improvements beyond the base neural network architecture.

In addition, to further assess the modular contribution of spatial integration, we applied the ABM module to velocity embeddings generated by other RNA velocity methods (e.g., scVelo) without retraining. Across datasets, this integration consistently improves velocity confidence (Fig. 6c), supporting that the performance gains are attributable, at least in part, to spatial refinement rather than solely the underlying model. For example, relative to scVelo adding the ABM module led to the following performance improvements (Fig. 6c): mouse brain (Stereo-seq, 0.9943 vs. 0.0677; $FDR < 1.0 \times 10^{-308}$), breast cancer (0.6920 vs. 0.0929; $FDR < 1.0 \times 10^{-308}$), chicken heart (0.9959 vs. 0.3550; $FDR < 1.0 \times 10^{-308}$), and mouse brain (HybISS, 0.9794 vs. 0.3166; $FDR < 1.0 \times 10^{-308}$). These consistent gains demonstrate that the ABM functions as a general-purpose enhancement, improving spatial coherence while preserving veloAgent's computational efficiency.

To improve clarity, we have revised Results ("veloAgent is scalable and computationally robust") and figure captions of Figure 6 to more explicitly highlight (i) the comparison between veloAgent with and without the spatial module and (ii) the cross-method augmentation experiments demonstrating the contribution of spatial information via the ABM module.

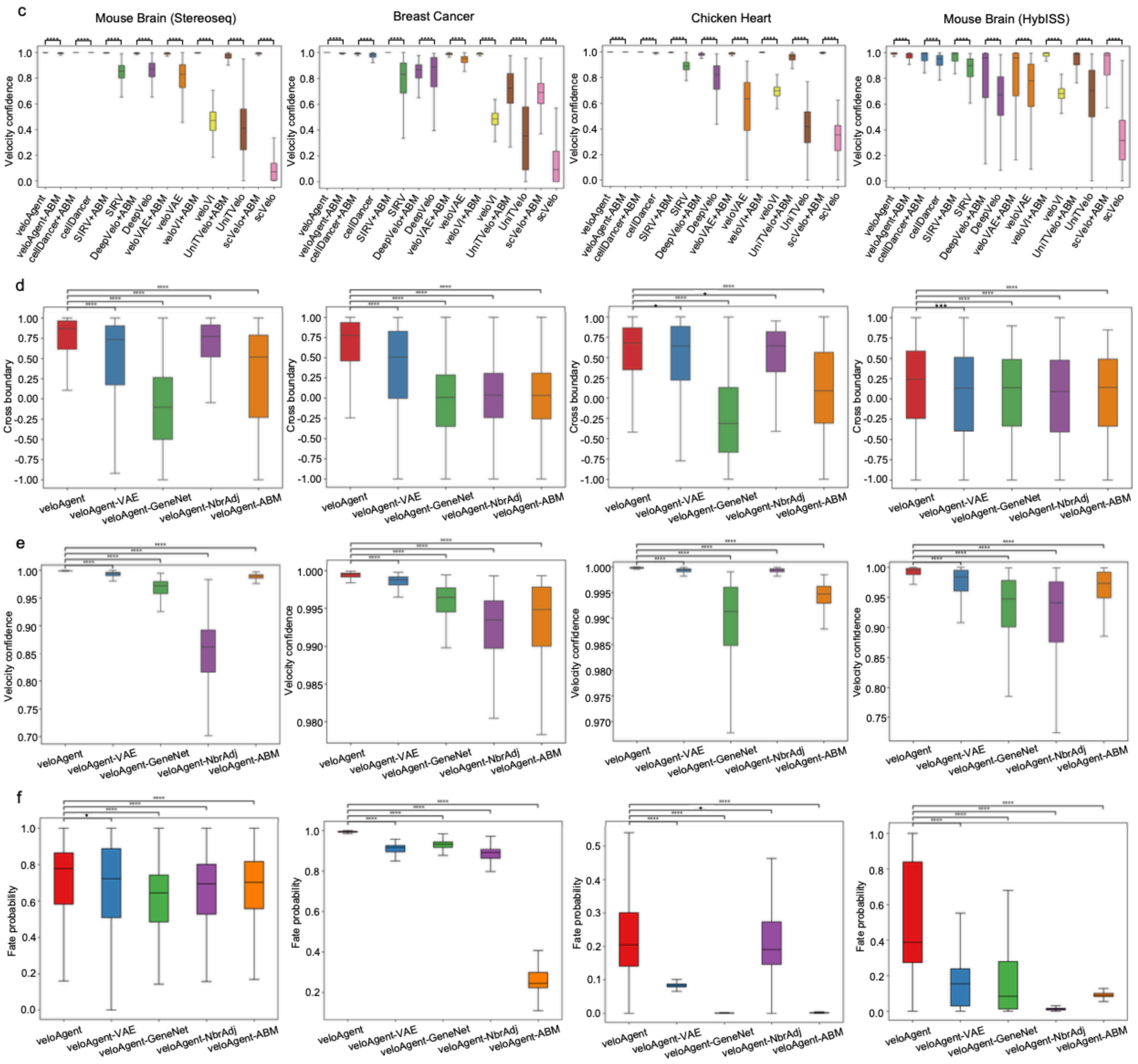

Figure 6 Ablation study of veloAgent. **c,** Comparative evaluation of velocity confidence scores before and after applying veloAgent's agent-based model (ABM) for spatial refinement on top of each method's temporal velocity estimates. Original estimates from each method are contrasted with their spatially adjusted counterparts, showing consistent improvement when spatial data is incorporated. **d-f,** To assess the contribution of key model components, an ablation study was performed by systematically removing each module from veloAgent, including the ABM module. **d,** Cross-boundary accuracy, **e,** velocity confidence and **f,** fate probability metrics across four configurations: full veloAgent, without latent-space smoothing, without gene-informed network architecture, without neighbour adjustment and without incorporating spatial transcriptomics data using agent-based modeling. Performance drops observed in ablated models validate the importance of each component. Error bars show interquartile range. Statistical significance calculated using Mann-Whitney U-test with FDR correction. Significance levels are indicated as follows: n.s, not significant (P ≥0.05); * (P<0.05); ** (P<0.01); *** (P<0.001); and **** (P<0.0001). Exact P-values can be found in Appendix Table S4.

Comment 3: Applying kinetic equations designed for single cells directly to multi-cell Visium spots may result in averaged dynamics that obscure true cell states.

Response: Thank you for raising this important point. We agree that Visium spots may contain multiple cells, and therefore the inferred dynamics represent aggregated transcriptional states rather than strictly single-cell kinetics. In this context, the estimated velocities should be interpreted as effective, spot-level dynamics that reflect the dominant transcriptional trends within each spatial location.

From a modeling perspective, applying kinetic formulations to spot-level data corresponds to operating on mixtures of cell states. Under a linear mixture approximation, the observed expression within a spot can be written as a weighted combination of underlying cellular states, and the corresponding temporal derivative (or velocity) follows the same linearity. As a result, the inferred velocity at the spot level can be interpreted as a weighted average of the underlying single-cell velocities. While this aggregation may attenuate magnitude, it preserves the principal directionality as long as the mixture is not composed of strongly opposing trajectories. This provides a theoretical basis for interpreting spot-level velocities as meaningful summaries of local dynamics.

To empirically assess this behavior, we evaluated veloAgent across multiple spatial transcriptomics platforms spanning different levels of granularity. For near single-cell resolution datasets (e.g., Stereo-seq and HybISS), where each spatial unit closely approximates individual cells, veloAgent produces spatially coherent and directionally consistent velocity fields that align with expected biological patterns. For spot-based platforms such as Visium, although each spot aggregates multiple cells, the inferred dynamics continue to capture consistent spatial structures, including coherent directional flows and biologically meaningful regional transitions. Importantly, we observe that the primary effect of aggregation is a smoothing of the velocity field rather than a disruption of its global directionality (Fig. 6d-f), supporting the above interpretation.

To further mitigate the loss of local structure due to aggregation, veloAgent incorporates an agent-based modeling (ABM) refinement module that leverages spatial neighborhood information. This module does not attempt to reconstruct single-cell dynamics, but instead enforces local consistency and enhances transitions between distinct regions, thereby reducing the blurring effect introduced by mixed-cell measurements.

We also note inherent limitations of this setting. In regions with highly heterogeneous cellular compositions, particularly where multiple divergent or opposing trajectories coexist within a single spot, the inferred velocity represents a composite signal and may not correspond to any individual cell state. In such cases, both magnitude and direction may be biased toward the dominant population or intermediate states. We have explicitly discussed this limitation in the Discussion of the revised manuscript.

These results indicate that, despite the inherent trade-off between spatial resolution and aggregation, veloAgent reliably captures biologically meaningful spatial trends in transcriptional dynamics across platforms. Accordingly, velocities inferred from Visium data should be interpreted at the level of regional or domain-level transitions rather than single-cell kinetics. We have revised the manuscript to clarify this interpretation, the underlying assumptions, and the associated limitations in the Discussion.

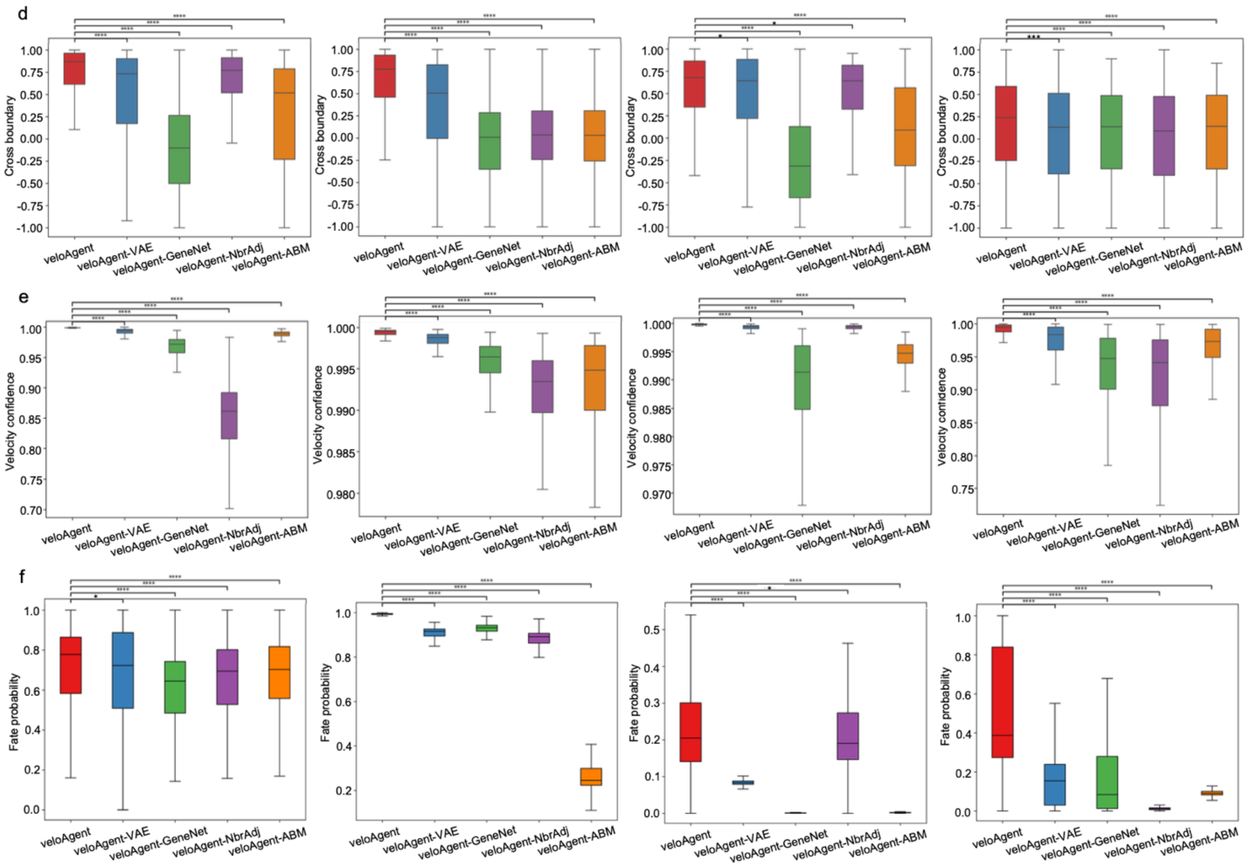

Figure 6 Ablation study of veloAgent. **d-f,** To assess the contribution of key model components, an ablation study was performed by systematically removing each module from veloAgent, including the ABM module. **d,** Cross-boundary accuracy, **e,** velocity confidence and **f,** fate probability metrics across four configurations: full veloAgent, without latent-space smoothing, without gene-informed network architecture, without neighbour adjustment and without incorporating spatial transcriptomics data using agent-based modeling. Performance drops observed in ablated models validate the importance of each component. Error bars show interquartile range. Statistical significance calculated using Mann-Whitney U-test with FDR correction. Significance levels are indicated as follows: n.s, not significant ($P \geq 0.05$); * ($P<0.05$); ** ($P<0.01$); *** ($P<0.001$); and **** ($P<0.0001$). Exact P-values can be found in Appendix Table S4.

Comment 4: Regarding the statement on Line 155 about qualitatively superior performance, it would be helpful to cite the specific figure panels that demonstrate this. Please specify the visual criteria or improvements the reader should focus on to support this claim.

Response: Thank you for this helpful suggestion. We have revised the manuscript to explicitly cite the relevant figure panels illustrating qualitative (Fig. 2a-h) and quantitative performance (Fig. 2i-k). Note this is now on line 174 of the revised manuscript.

As per your suggestion, we now specify in the Results section the key visual features supporting the qualitative assessment, including more coherent and continuous velocity fields within spatial domains, sharper alignment of transitions with anatomical or regional boundaries, and reduced spurious directional fluctuations. These features are highlighted by red circles in panels 2a-d to guide the qualitative evaluation of veloAgent.

Comment 5: Since current benchmarks primarily evaluate gene expression similarity, it would be valuable to incorporate metrics that specifically assess spatial accuracy. Using spatially-aware metrics (whether existing or newly proposed) would strengthen the evaluation of the model's spatial performance.

Response: Thank you for this important comment. We agree that evaluating accuracy from a spatial perspective is essential for assessing spatially informed RNA velocity methods. In our study, we incorporated spatially aware evaluation metrics, including spatial velocity confidence and spatial fate probability, which directly quantify spatial accuracy (Appendix Figure S9).

Specifically, these metrics assess whether inferred velocity vectors are consistent across neighboring spatial locations, thereby capturing tissue-level coherence of cell-state transitions rather than relying solely on transcriptomic similarity. In this way, they provide a spatially grounded evaluation of whether the inferred dynamics align with local tissue organization.

These spatial metrics are reported in the Results section ("Spatially informed RNA velocity reveals tissue-coherent cell-state transitions") and presented in Figure 3 and Appendix Figure S9, where they are used to quantify the extent to which veloAgent improves spatial RNA velocity relative to competing methods. To improve clarity, we have revised the manuscript in both the Results and Methods ("Benchmarking") sections to explicitly highlight these as spatial performance metrics and to distinguish them from conventional transcriptomic similarity-based evaluations.

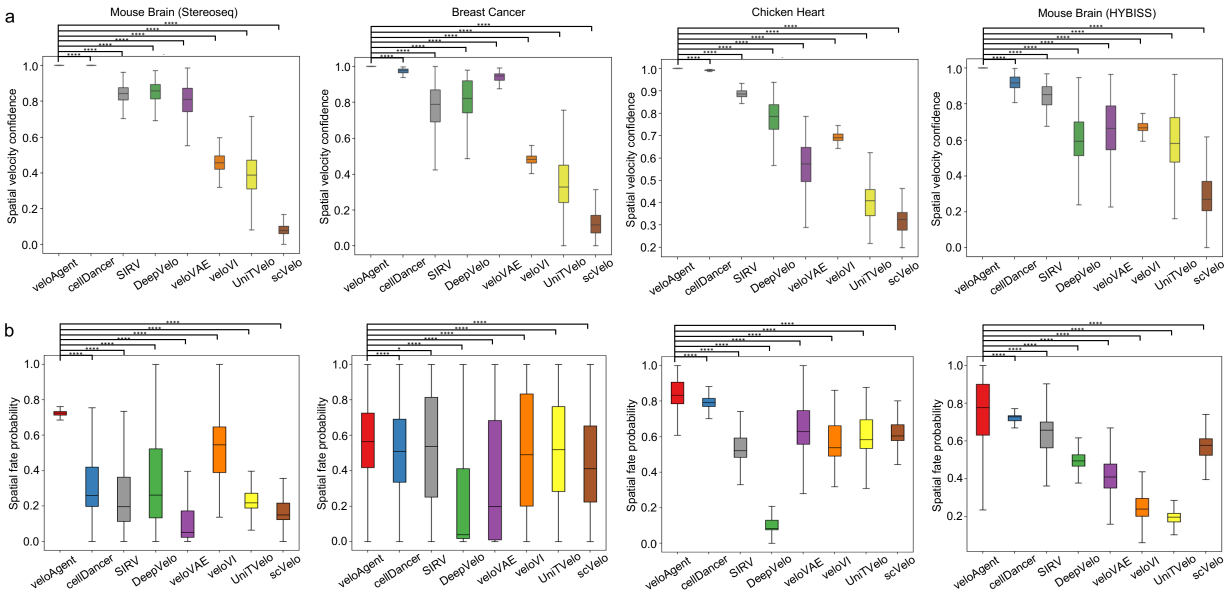

Appendix Figure S9: Evaluation of RNA velocity quality on spatial map. a, Spatial velocity confidence (mean velocity confidence across sub-grids of dataset) comparison between veloAgent and all other methods. **b,** Spatial fate probability (fate probability calculated based off spatial mapping) score evaluations between veloAgent and all other methods. veloAgent's performance gap from other methods and its consistency emphasizes the importance of spatial context in RNA velocity inference. Error bars show interquartile range. Statistical significance calculated using Mann-Whitney U-test with FDR correction. Significance levels are indicated as follows: n.s, not significant (P ≥0.05); * (P<0.05); ** (P<0.01); *** (P<0.001); and **** (P<0.0001).

Comment 6: Visualizing velocity arrows directly on the spatial map can be potentially misleading, as it may imply physical migration rather than differentiation state changes.

Response: Thank you for this important observation. You have noted that visualizing velocity vectors on spatial maps may be misinterpreted as indicating physical cell migration rather than transcriptional state transitions. In response, the manuscript has been revised to explicitly clarify that the projected velocity vectors represent transcriptional state changes inferred from RNA velocity and do not correspond to physical movement of cells within the tissue. This clarification has been added in both the Results ("Spatially informed RNA velocity reveals tissue-coherent cell-state transitions") and the corresponding figure caption (Fig. 3). These revisions are intended to eliminate potential ambiguity and ensure that the visualization is interpreted in the correct biological context.

Reviewer 2

General Comments: RNA velocity inference has garnered significant attention, prompting the development of numerous computational frameworks. However, this study presents an innovative deep learning framework veloAgent that addresses several key limitations in current velocity

analyses. veloAgent is one of the first methods that can infer spatially resolved RNA velocity through deep integration of spatial transcriptomics data into the velocity inference process via an Agent-Based Model (ABM). This integration enables the inference of cell trajectories that are anatomically more plausible and the influence of the tissue microenvironment. To overcome the scalability bottlenecks of existing methods, veloAgent adopts a gene-centric model architecture whose computational complexity does not increase drastically with the number of cells. More importantly and interestingly, this study introduces the field's first in silico perturbation module, which transforms RNA velocity from a descriptive tool into a causal inference system capable of identifying key regulatory factors. By incorporating prior knowledge of gene interactions into neural network, veloAgent also improves the model's biological interpretability. The authors demonstrate the superior performance of veloAgent on several datasets with varying technologies and through a solid comparison analysis with other methods.

Overall, this new approach represents a significant advance over existing methods, and serves as a unique and useful tool for inferring spatially resolved RNA velocity and manipulating spatial velocity to simulate regulatory interventions. However, I still have a few comments, especially on the method details, that should be addressed.

Response: Thank you for your thoughtful and encouraging feedback. We appreciate your recognition of the innovation and utility of veloAgent, particularly its integration of spatial transcriptomics through the ABM framework and its capability for in silico perturbation analysis.

In response to your comments regarding methodological detail, we have revised the manuscript to improve clarity, robustness, and reproducibility. Specifically, we performed sensitivity analyses for key hyperparameters (τ and σ_{ABM}) to assess the stability of the model and demonstrate that performance is not driven by specific parameter choices. We also conducted additional ablation studies to quantify the contributions of the density weighting and STRING-based prior, thereby clarifying the role of each component in the overall framework. In addition, we have added a dedicated hyperparameter section in the Methods to provide a complete and transparent description of parameter settings and training procedures, facilitating reproducibility.

These revisions directly address the need for clearer methodological description and more rigorous evaluation of model components, and we have updated the manuscript accordingly.

Comment 1: It is important to performance the sensitive analysis of the regularization parameters lambda and tau in the overall loss function. High parameter values will likely lead to over-smoothed RNA velocity across cell neighbors. A potential problem may also exist in the ABM-based model of spatial velocity. While this is challenging problem in the field, is there any suggestion to balance the similarity and sharpness of a cell's RNA velocity against its neighbors?

Response: We thank you for pointing out this critical problem and we fully agree that sensitivity analysis of the regularization parameters is essential, especially given the risk that overly large values may produce over-smoothed RNA velocity estimates across neighboring cells.

Upon revisiting the manuscript, we realized that the inclusion of the hyperparameter λ in the loss function in Eq. 8 was inaccurate. In our implementation, the VAE and the neural network module are trained sequentially as separate components rather than through a single end-to-end optimization. Therefore, there is no weighting hyperparameter controlling the relative contribution of the neural network loss during training. We have removed λ from the loss formulation in the revised manuscript so that it accurately reflects the actual training procedure, and we have also updated Figure 1 accordingly. The updated Eq. 8 is provided below:

$$\boldsymbol{\mathcal{L}} = \boldsymbol{\mathcal{L}}_{VAE} + \boldsymbol{\mathcal{L}}_{NN} + \boldsymbol{\tau}\boldsymbol{\mathcal{L}}_{NBR} \quad (8)$$

Regarding τ, this parameter is used during the neighborhood refinement step, where the neural network-inferred kinetic parameters are further updated to refine the velocity estimates. In this setting, τ controls the strength of the additional regularization introduced during refinement, thereby influencing the balance between preserving local similarity across neighboring cells and maintaining sharper, cell-specific velocity patterns.

Following your suggestion, we performed a sensitivity analysis for τ and evaluated performance using cross-boundary correctness and velocity confidence, two widely used metrics for RNA velocity evaluation (Appendix Figure S19). We found that $\tau = 0.2$ gave the best overall performance. We have now included this default value in the hyperparameter section of the Methods.

For the ABM-based spatial velocity model, we also carried out a sensitivity analysis of σ_{ABM} (Appendix Figure S20). We note that there was inaccurate description of the ABM hyperparameters in the original version of our manuscript. In our implementation, there is no separate λ_{ABM}, rather the complementary term is set as $\lambda_{ABM} = 1 - \sigma_{ABM}$. We have updated and clarified this in our revised Methods section. In our sensitivity analysis, velocity confidence remained high across tested values (Appendix Figure S20b), while cross-boundary correctness was generally similar but performed slightly better at $\sigma_{ABM} = 0.7$ across datasets (Appendix Figure S20a). We have therefore included $\sigma_{ABM} = 0.7$ as the default value in the newly added "Hyperparameters" subsection in the revised Methods.

More broadly, we agree with the reviewer that balancing similarity and sharpness is an important challenge in spatial RNA velocity modeling. In our framework, this balance is controlled by the neighbour refinement strength τ and the ABM weighting parameter σ_{ABM}. Empirically our sensitivity analyses suggest that moderate regularization provides the best trade off, allowing the

model to incorporate local neighbourhood and spatial coherence without excessively over-smoothing cell-specific velocity structure (Appendix Figure S19 and S20).

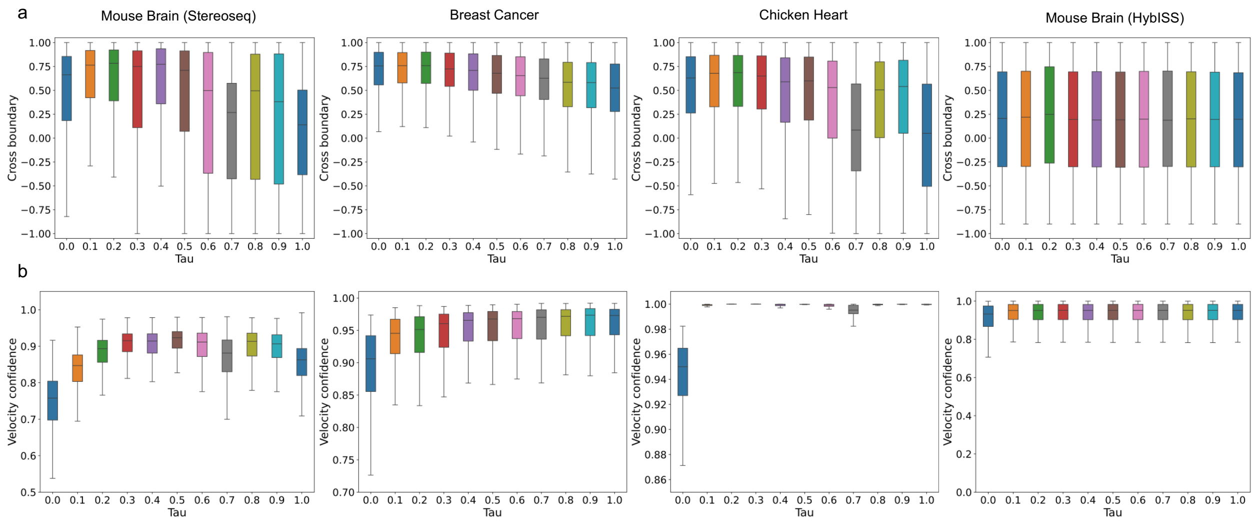

Appendix Figure S19: Quantitative performance of veloAgent using different τ values. a, Cross-boundary scores across τ values ranging from 0-1 (inclusive). This metric measures the correctness of predicted transitions across annotated cluster boundaries, based on ground-truth developmental labels. Higher scores reflect better agreement with known trajectories. **b,** Velocity confidence scores across τ values ranging from 0-1 (inclusive). This metric quantifies how well predicted velocities align with local gene expression structure. Higher scores indicate more reliable predictions. $\tau = 0.2$ is the default value used in veloAgent. Error bars show interquartile range.

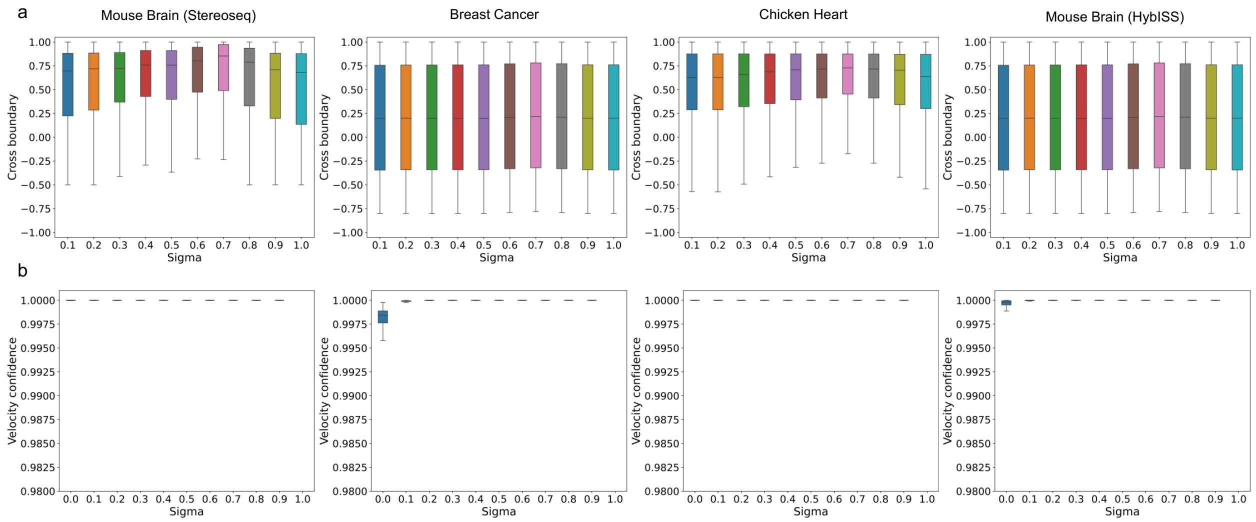

Appendix Figure S20: Quantitative performance of veloAgent across different σ_{ABM} values. a, Cross-boundary scores for σ_{ABM} values ranging from 0-1 (inclusive). This metric measures the correctness of predicted transitions across annotated cluster boundaries based on ground-truth developmental labels. Higher scores reflect better agreement with known trajectories. **b,** Velocity confidence scores for σ_{ABM} values ranging from 0-1 (inclusive). This metric quantifies how well predicted velocities align with local

gene expression structure. Higher scores indicate more reliable predictions. $\sigma_{ABM} = 0.7$ is the default value used in veloAgent. Error bars show interquartile range.

Comment 2: In the ABM part, please clarify the meaning of the variable in equation 10 and how to compute it. In equation 12, Is DV_i the velocity calculated after Phase 3? Should DW_i be also added in the second term related to the cell neighbors? How was the performance if the density weight was not included in equation 12?

Response: Thank you for the opportunities for us to answer these clarification questions. We have revised the manuscript to clarify the meaning of the variables used in the ABM formulation. Specifically, we now explicitly define ρ_i as in Eq. 10 as the local cell density for cell i, computed as the number of neighbouring cells with the defined spatial radius. Regarding Eq. 12, RV_i denotes the velocity of cell i after phase 3 of the pipeline. We have also clarified this in the revised Methods section.

With respect to the density weight DW_i, it is applied only in the first term of Eq. 12 because this term represents the intrinsic contribution of the focal cell, whereas the second term aggregates the influence of neighboring cells through distance and expression similarity weights. The neighbor term already has separate weights for proximity and expression similarity, so adding DW_i there may double-count local structure.

To evaluate the importance and necessity of the density weight, we performed an additional ablation analysis in which DW_i was removed from the formulation. Performance was assessed using two widely used metrics: cross-boundary direction and velocity confidence. We observed that removing the density weight reduced cross-boundary direction accuracy, indicating weaker directional consistency across lineage transitions (Appendix Figure S18a). In contrast, velocity confidence remained high in both settings (Appendix Figure S18b). We believe this occurs because, without the density weighting, neighbor velocities dominate the update step and produce an overly smoothed velocity field. These observations support the inclusion of the density weighting term to maintain biologically meaningful directional transitions and prevent over smoothing of velocities of neighbouring cells.

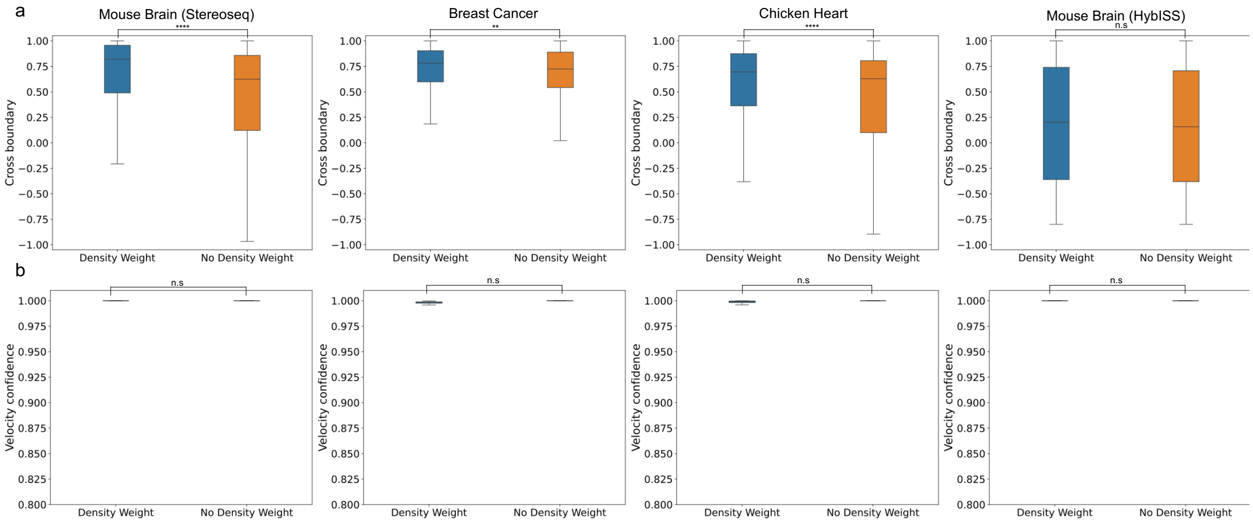

Appendix Figure S18: Quantitative performance of veloAgent with and without the density weight (DW) in the ABM. a, Cross-boundary scores of veloAgent with and without the density weight (DW) in the ABM. This metric measures the correctness of predicted transitions across annotated cluster boundaries based on ground-truth developmental labels. Higher scores reflect better agreement with known trajectories. **b,** Velocity confidence scores of veloAgent with and without the density weight (DW). This metric quantifies how well predicted velocities align with local gene expression structure. Higher scores indicate more reliable predictions. Error bars show interquartile range. Statistical significance calculated using Mann-Whitney U test with FDR correction. Significance levels are indicated as follows: n.s, not significant ($P \geq 0.05$); * ($P<0.05$); ** ($P<0.01$); *** ($P<0.001$); and **** ($P<0.0001$).

Comment 3: Using prior gene interaction information appears to reduce computational complexity, but the fully-connected approach for genes without prior information weakens this advantage. Can the authors clarify the proportion of genes without prior information? In addition, how many genes are used in this Phase? What is the threshold of confidence scores when selecting gene-gene interactions from STRING database?

Response: We thank the reviewer for raising this important comment. We would like to clarify that incorporating prior gene interaction information not only reduces the computational burden compared with a fully connected formulation, but also allows the model to leverage known biological relationships to better estimate the transcriptional dynamics parameters, including the α, β and γ rates.

The exact proportion of genes with prior interaction information varies across datasets and species, since each dataset contains a different gene set and STRING coverage differs by organism. We computed these proportions for the main datasets used in our study. Specifically, 99.2% of genes in the mouse brain Stereo-seq dataset, 99.1% in the mouse brain HyBISS dataset, 96.4% in the chicken heart dataset, and 41.0% in the breast cancer dataset were present in STRING (Appendix Figure S17a). Overall, this shows that STRING covers the vast majority of genes in the non-human datasets considered here, while coverage is substantially lower for the breast cancer dataset, which is the only human dataset included in our analysis.

We also clarify that, in our main experiments, we did not apply a confidence score threshold when selecting gene-gene interactions from STRING. Instead, we included all genes and interactions available in STRING in order to incorporate as much prior biological knowledge as possible. This choice is consistent with prior work that similarly uses broad prior interaction information rather than aggressively filtering for only high-confidence edges (Raghavan *et al*, 2025). We have clarified this in the revised Methods.

Nevertheless, to directly evaluate this design choice, we performed an additional comparison between using all available STRING interactions and using only interactions passing a confidence-based filter. Specifically, for the filtered setting, we retained only interactions with a combined STRING score of 400 or higher, as this corresponds to medium-confidence interactions (Szklarczyk *et al*, 2023). This removed approximately 90% of interactions and retained only the top 10% of interactions in the database. We found that using all available prior interactions led to better performance than restricting the graph to only high-confidence interactions (Appendix Figure S17b, c). We believe this may be because applying a strict confidence threshold removes too many potentially informative gene-gene connections, limiting the gene-gene neural network model capacity.

These findings, together with our earlier ablation results, further emphasize that incorporating prior interaction information leads to improved performance compared with using a fully connected network, thereby supporting the benefit of this design choice.

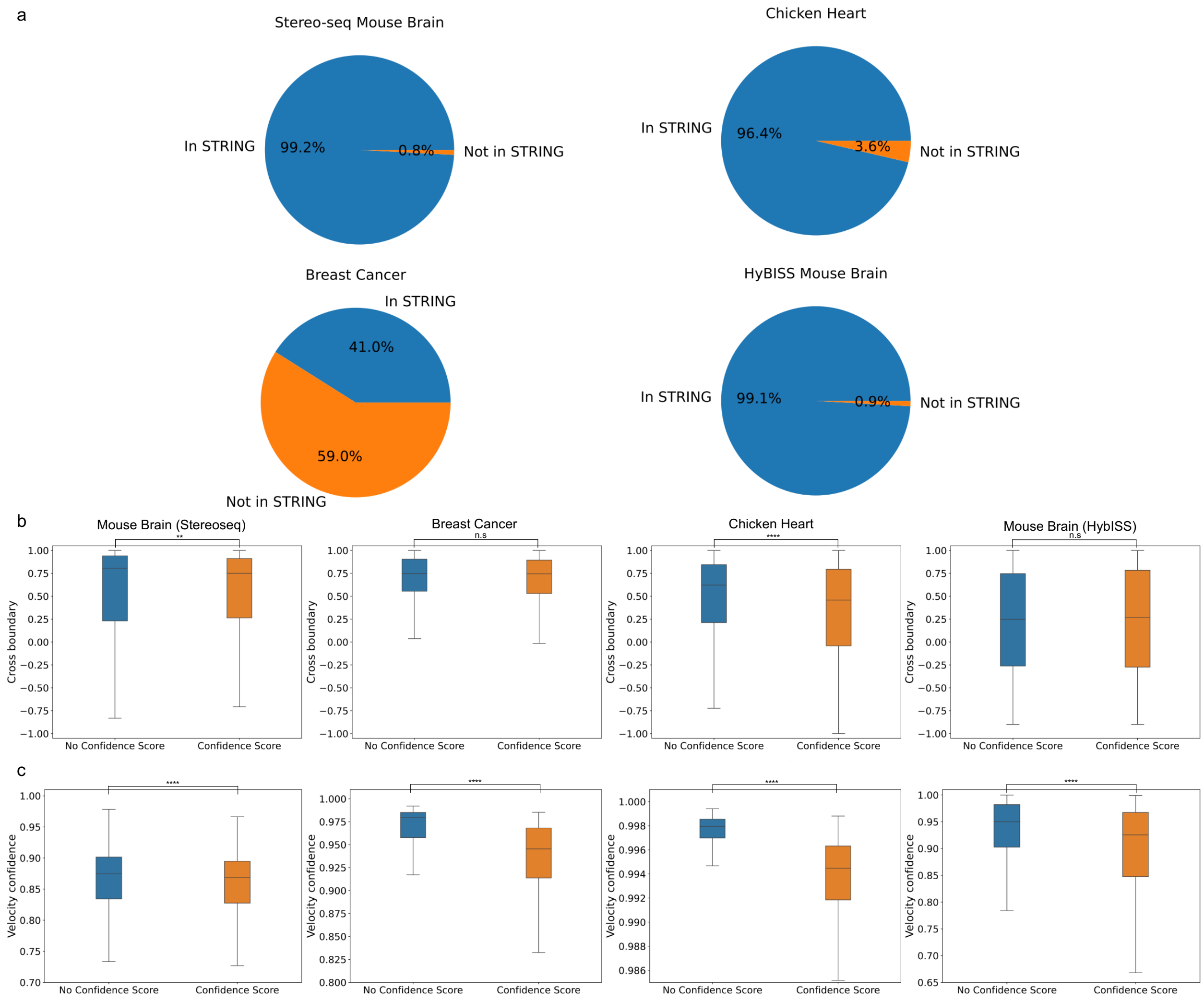

Appendix Figure S17: Evaluation of incorporating gene-gene interaction prior from STRING. a, Pie charts show the proportion of genes in each dataset that are present in STRING and therefore incorporated as prior knowledge in the gene-gene neural network. **b,** Cross-boundary scores of veloAgent with and without applying confidence score filtering (combined score greater than 400; medium confidence or better) to restrict gene-gene interactions in the neural network. This metric measures the correctness of predicted transitions across annotated cluster boundaries based on ground-truth developmental labels. Higher scores reflect better agreement with known trajectories. **c,** Velocity confidence scores of veloAgent with and without applying confidence score filtering (combined score greater than 400; medium confidence or better) to restrict STRING-derived gene-gene interactions in the neural network. This metric quantifies how well predicted velocities align with local gene expression structure. Higher scores indicate more reliable predictions. Error bars show interquartile range. Statistical significance calculated using Mann-Whitney U test with FDR correction. Significance levels are indicated as follows: n.s, not significant ($P \geq 0.05$); * ($P<0.05$); ** ($P<0.01$); *** ($P<0.001$); and **** ($P<0.0001$).

Comment 4: Method details of hyperparameter settings are missing. For example, the learning rate, optimizer choice, batch size, and the latent dimension size of the VAE, the radius r, the sigma and the lambda in the ABM method. Please check all hyperparameters and clarify the default settings.

Response: Thank you for highlighting the need for clearer reporting of hyperparameter settings. As per your suggestion, the Methods section has been revised to comprehensively report all relevant hyperparameters used in each module of veloAgent. Specifically, the revised manuscript now includes optimizer choice, learning rates, weight decay, number of training iterations, VAE latent dimension size, neighborhood size, τ for controlling neighbour refinement and all ABM-related parameters (r, σ_{ABM}, $\tau_{spatial}$). These parameters are organized in a dedicated "Hyperparameters" subsection in the revised Methods to improve clarity and reproducibility.

Comment 5: What is the meaning of the colors in the gene velocity plots such as Fig. 2e and Fig. 3f? The font size of GO terms in several figure panels are very small. It would be helpful to increase it.

Response: Thank you for the comment. In Fig. 2e-h and Fig. 3f, the colors represent the magnitude of gene-specific RNA velocity. Lighter colors (green to yellow) indicate higher velocity magnitudes, whereas darker colors (purple) indicate lower magnitudes. To improve clarity, we have added an explicit description of the color scale in the figure captions of Figures 2 and 3. In addition, we have increased the font size of the GO term labels in Figures 4b, 4e, 4h, and 5d to enhance readability, as suggested.

Reviewer 3

General Comments: The manuscript presents veloAgent, a hybrid deep learning and agent-based framework for estimating RNA velocity by integrating gene expression data with prior knowledge of gene-gene interactions and spatial context. The method involves several stages: a variational autoencoder encodes spliced and unspliced mRNA counts into a latent space; a constrained neural network infers cell- and gene-specific kinetic parameters; and an agent-based module refines velocity vectors using transcriptional similarity, local density, and intercellular distance. The authors also demonstrate the use of in silico perturbations to assess gene-level contributions to trajectory directionality and highlight the model's scalability across large and multi-batch datasets. Notably, the authors perform ablation studies on individual components of the model-a practice that is rare in similar studies.

Response: Thank you for your thoughtful and encouraging feedback on our manuscript. We greatly appreciate your clear summary of veloAgent and your recognition of its hybrid deep learning and agent-based design, its scalability, and its ability to support in silico perturbation analysis. We are also especially grateful that you highlighted the inclusion of ablation studies on individual model components, as we agree that this is an important aspect for understanding the contribution of each part of the framework.

In response to your comments, we have revised the manuscript to further improve clarity on several methodological aspects. In particular, we provided additional explanation of how prior gene-gene interaction information from STRING is incorporated and clarified the modeling choices associated with its use. We also expanded the discussion of the benefits of using VAE-derived embeddings, including their role in denoising and dimensionality reduction, and supported this design choice with an additional ablation comparing embeddings against raw spliced/unspliced counts. In addition, we performed sensitivity analyses for the key hyperparameters τ and σ_{ABM}, and incorporated these results into the revised manuscript to improve clarity and reproducibility.

A detailed, point-by-point response to your specific comments is provided below. Thank you again for your valuable feedback and support.

Comment 1: (Line 612) The description of "sparse connections informed by gene-gene interaction data" lacks implementation details. Please clarify how the interaction data are incorporated into the model architecture.

Response: We thank the reviewer for highlighting the need for additional implementation details regarding how we create sparse connections informed by gene-gene interaction data. We have revised the manuscript to clarify how the gene-gene interaction data from the STRING database are incorporated into the neural network architecture in the Methods "veloAgent framework". Specifically, we now explain that the first and second hidden layers are each of size *G* (the number of genes). The connections between these layers are sparse connection defined by gene-gene interactions from the STRING database, where a connection is introduced between two gene nodes in the model if an interaction between those genes are reported in STRING. To maximize incorporation of prior biological knowledge, we include all reported interactions and do not filter edges based on STRING confidence scores. Importantly, this procedure is consistent with approaches used in prior work (Raghavan *et al.*, 2025), supporting our modeling choices.

Comment 2: (Line 616) Is it reasonable to connect genes without known interactions to all other gene nodes? This design seems biologically implausible and may introduce noise.

Response: We thank the reviewer for raising this important point. We agree that connecting genes without known interactions to all other gene nodes is biologically implausible and may introduce additional noise. However, we chose this design to avoid excluding genes that lack documented interactions in current databases but may still play functional roles in the regulatory network. By allowing these genes to be fully connected, the model retains the ability to learn potential interactions directly from the data rather than discarding these genes entirely. This strategy ensures that genes with incomplete prior knowledge are not systematically ignored during training.

Importantly, the proportion of genes without prior interaction information is limited in most datasets (Appendix Figure S17a), meaning that the overall network structure remains largely constrained by biologically informed sparse connections. In addition, these connections are not fixed but are learned during training, allowing the model to down-weight spurious interactions and mitigate potential noise. We have clarified this point in the Methods section. We also show in our ablation results that our approach using gene interaction priors—combining structured sparse connections for genes with known interactions and fully connected modeling for genes without prior information—leads to higher performance than using a fully connected neural network (Figure 6d–f).

We note that similar approaches have been adopted in prior work where biological interaction networks are used as structural priors but are supplemented with learnable connections to account for incomplete knowledge (Raghavan *et al.*, 2025). To acknowledge this limitation more clearly, we have added a discussion of this design choice and its potential implications in the revised Discussion section.

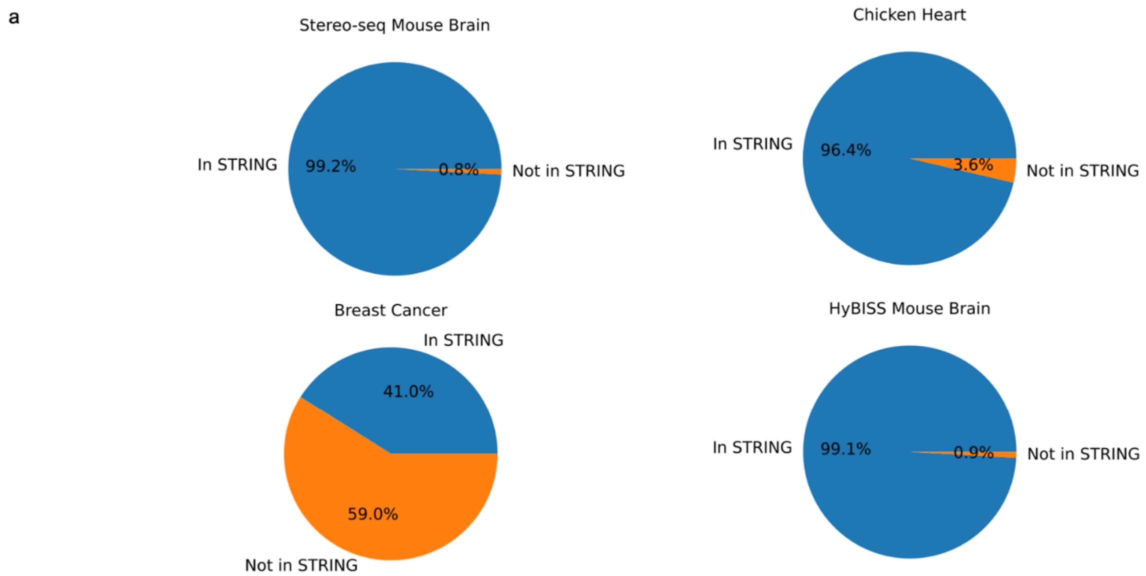

Appendix Figure S17: Evaluation of incorporating gene-gene interaction prior from STRING. a, Pie charts show the proportion of genes in each dataset that are present in STRING and therefore incorporated as prior knowledge in the gene-gene neural network.

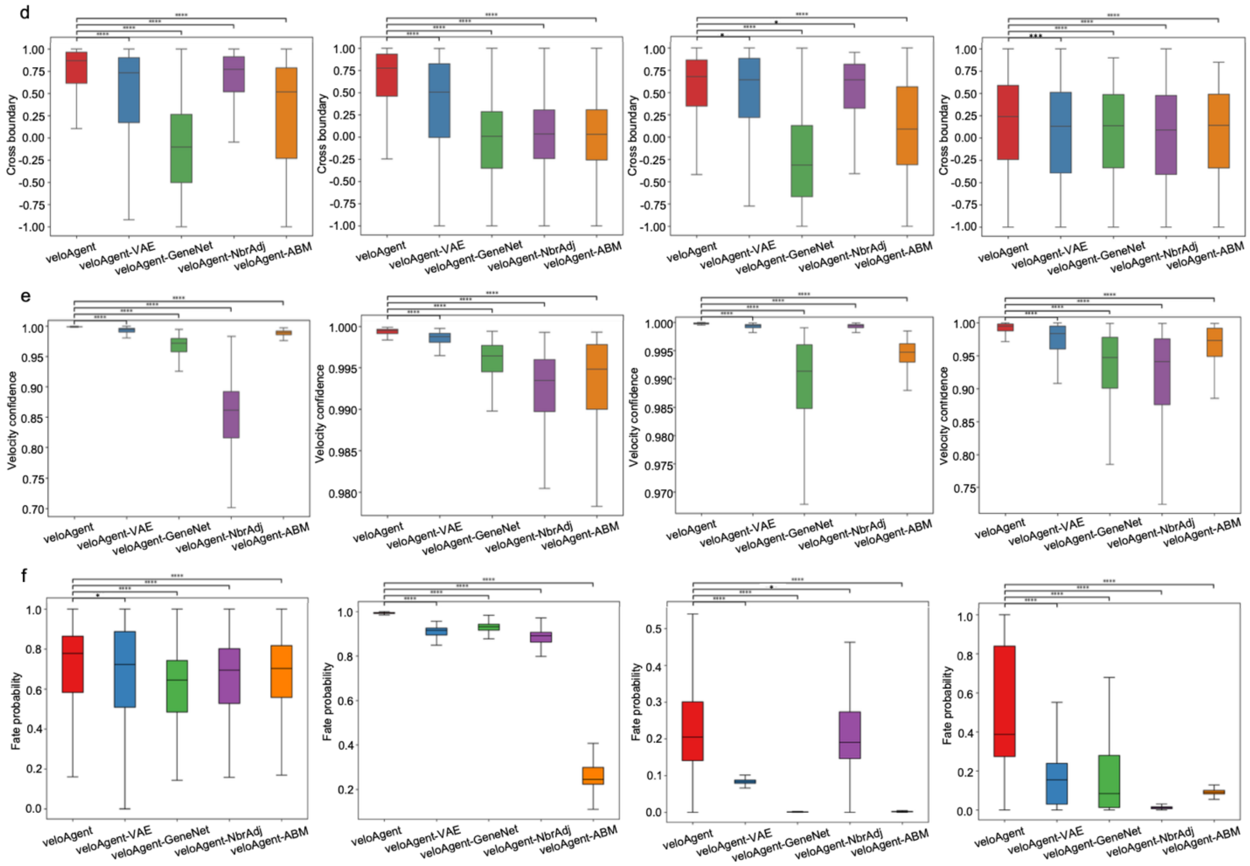

Figure 6 Ablation study of veloAgent. **d-f,** To assess the contribution of key model components, an ablation study was performed by systematically removing each module from veloAgent, including the ABM module. **d,** Cross-boundary accuracy, **e,** velocity confidence and **f,** fate probability metrics across four configurations: full veloAgent, without latent-space smoothing, without gene-informed network architecture, without neighbour adjustment and without incorporating spatial transcriptomics data using agent-based modeling. Performance drops observed in ablated models validate the importance of each component. Error bars show interquartile range. Statistical significance calculated using Mann-Whitney U-test with FDR correction. Significance levels are indicated as follows: n.s, not significant ($P \geq 0.05$); * ($P<0.05$); ** ($P<0.01$); *** ($P<0.001$); and **** ($P<0.0001$). Exact P-values can be found in Appendix Table S4.

Comment 3: (Line 634) The loss function appears similar to that of CellDancer, but no citation is provided. Please acknowledge prior work if applicable.

Response: We thank the reviewer for pointing this out. We have now explicitly cited cellDancer in the revised manuscript and clarified that our loss formulation follows this prior work. Note that this line in the revised manuscript is now line 705.

Comment 4: (Line 634) CellDancer uses raw spliced/unspliced counts as input, whereas this work uses VAE-derived embeddings. Given potential information loss from VAE compression, does this

design offer advantages over CellDancer? Note that CellDancer could also be extended with gene-gene interaction priors.

Response: We thank the reviewer for this insightful comment. We agree that cellDancer uses raw spliced and unspliced counts as input, whereas our method uses VAE-derived embeddings. The motivation for this design is that the VAE provides both dimensionality reduction and denoising, which can help extract a more compact and robust representation of the transcriptional state before modeling gene-gene interactions. This is particularly useful in single-cell data, where raw counts are often high-dimensional and noisy (Lopez *et al*, 2018).

To directly evaluate whether this design offers an advantage, we performed an ablation study in which we replaced the VAE embeddings with raw spliced/unspliced counts as input to the gene-gene neural network. We assessed performance using cross-boundary correctness and velocity confidence, two of the most widely used metrics for RNA velocity evaluation. We found that using raw counts led to similar or often decreased performance compared with using the VAE-derived embeddings (Appendix Figure S15). These results suggest that, despite the possibility of some compression-related information loss, the benefits of denoising and low-dimensional representation outweigh this drawback in practice, leading to improved downstream velocity estimation.

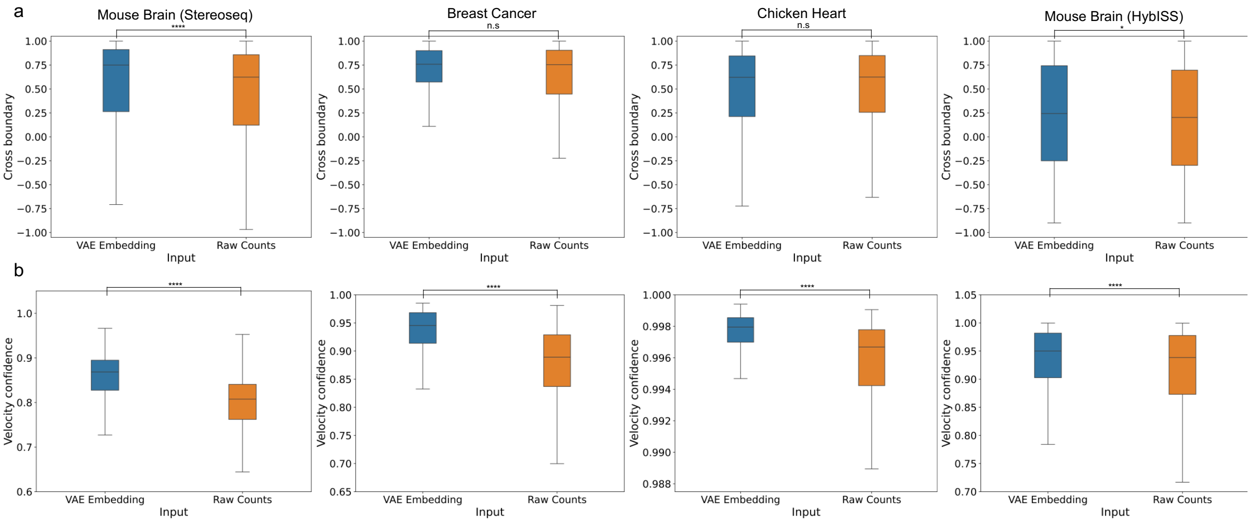

Appendix Figure S15: Quantitative performance of veloAgent with and without using VAE. a, Cross-boundary scores of veloAgent using the VAE embedding as input to the gene-gene neural network, compared with using raw unspliced and spliced counts as input. This metric measures the correctness of predicted transitions across annotated cluster boundaries based on ground-truth developmental labels. **b,** Velocity confidence scores of veloAgent using the VAE embedding as input to the gene-gene neural network, compared to using raw unspliced and spliced counts as input. This metric quantifies how well predicted velocities align with local gene expression structure. Higher scores indicate more reliable predictions. Error bars show interquartile range. Statistical significance calculated using Mann-Whitney U-test with FDR correction. Significance levels are indicated as follows: n.s, not significant ($P \geq 0.05$); * ($P<0.05$); ** ($P<0.01$); *** ($P<0.001$); and **** ($P<0.0001$).

Comment 5: (Line 651) How were the hyperparameters λ and τ in loss function (8) chosen? Their selection is critical given the multi-component loss.

Response: We thank the reviewer for highlighting this point. Upon revisiting the manuscript, we realized that the inclusion of the hyperparameter λ in the loss function in Eq. 8 was inaccurate. In our implementation, the VAE and the neural network module are trained sequentially as separate components rather than through a single end-to-end optimization. Consequently, there is no weighting hyperparameters controlling the relative contributions of the neural network loss during training. We have therefore removed λ from the loss formulation in the manuscript to accurately reflect the actual training procedure. We have also updated our Figure 1 to reflect this change. The updated Eq. 8 is provided below:

$$\boldsymbol{\mathcal{L}} = \boldsymbol{\mathcal{L}}_{VAE} + \boldsymbol{\mathcal{L}}_{NN} + \boldsymbol{\tau}\boldsymbol{\mathcal{L}}_{NBR} \qquad (8)$$

Regarding τ, this parameter is used during the neighborhood refinement step, where the neural network inferred kinetic parameters are updated to refine the velocity estimates. In this context, τ controls the strength of the additional regularization introduced during refinement. By default, we set τ=0.2, which provided stable convergence and consistent performance across datasets. We have added the default value of τ=0.2 in our newly added "Hyperparameters" subsection in the Methods. We showed that in a τ sensitivity analysis that τ=0.2, led to the best model performance across all datasets (Appendix Figure S19).

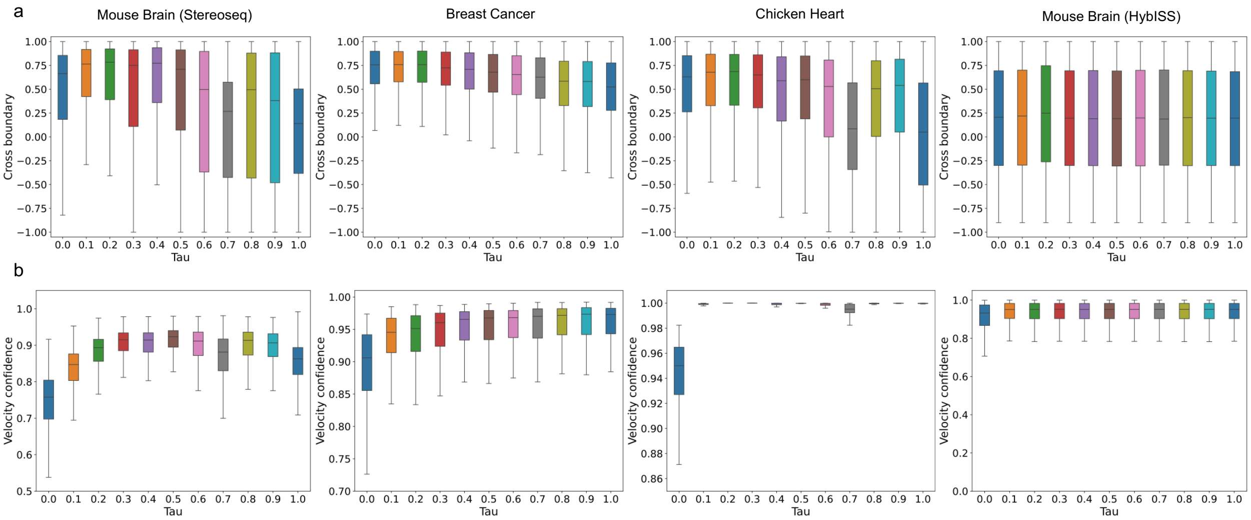

Appendix Figure S19: Quantitative performance of veloAgent using different τ values. a, Cross-boundary scores across τ values ranging from 0-1 (inclusive). This metric measures the correctness of predicted transitions across annotated cluster boundaries, based on ground-truth developmental labels. Higher scores reflect better agreement with known trajectories. **b,** Velocity confidence scores across τ values ranging from 0-1 (inclusive). This metric quantifies how well predicted velocities align with local

gene expression structure. Higher scores indicate more reliable predictions. $\tau = 0.2$ is the default value used in veloAgent. Error bars show interquartile range.

Comment 6: (Lines 666-689) Several variables are not clearly defined, e.g., ρ in Equation (10). Please specify their definition.

Response: We thank the reviewer for highlighting the lack of clarity in this section. We have revised the manuscript to explicitly define these variables. In particular, ρ_i in Eq. 10 refers to the local density of cell *i,* calculated as the number of neighbouring cells within the predefined spatial spot radius. This definition has been added directly in the text when introducing Eq. 10.

To further improve clarity, we also clarified that x_i in Eq. 9 denotes the gene expression profile of cell i. In addition, we now introduce the abbreviations RV, DW, SW and SpW directly in the text where they first appear, rather than only within the equations. These changes make the notation in this section more explicit and easier to follow.

Comment 7: (Lines 666-689) The term "agent-based module" may be misleading, as the module only performs post-hoc velocity refinement and does not involve autonomous agents. Consider using a more descriptive name.

Response: You raise an important point regarding the use of the term "agent-based." It is agreed that the current module does not constitute a full agent-based simulation framework in which agents evolve over multiple time steps with autonomous state transitions. Instead, it functions as a post-hoc refinement step applied to initially inferred RNA velocities.

The term "agent-based" is used here in the context of classical agent-based modeling, where individual entities—in this case, cells—are treated as agents whose states are updated based on interactions with their local environment according to predefined rules. In veloAgent, each cell's velocity is refined through explicit modeling of local interactions with neighbouring cells, incorporating spatial proximity and transcriptional similarity. The key defining characteristic of this formulation is that updates are performed at the level of individual cells based on local interaction rules, which is a central principle of agent-based modeling, even in the absence of multi-step temporal simulation.

It is acknowledged that this represents a simplified and single-step instantiation of the agent-based modeling paradigm rather than a full dynamical system. Nevertheless, the term "agent-based" is retained because the refinement mechanism is explicitly formulated as rule-based updates over interacting cellular entities, thereby preserving the core modeling abstraction of ABM.

To avoid potential ambiguity, the manuscript has been revised to clarify this scope in the Methods section when introducing the module, explicitly stating that the ABM refers to a localized,

interaction-based refinement rather than a full multi-step simulation framework. This clarification has also been added to the Discussion section.

Comment 8: (Lines 666-689) How should the hyperparameters σ_ABM, λ_ABM, and neighborhood radius r be selected? In particular, how is r determined for different spatial transcriptomics technologies?

Response: Thank you for these clarification questions. We would first like to clarify that there was a mistake in our description of the ABM hyperparameters. In our implementation, we set $\lambda_{ABM} = 1 - \sigma_{ABM}$. For improved clarity we have corrected in in our revised Methods section by replacing λ_{ABM} with $1 - \sigma_{ABM}$ to ensure that the hyperparameter definition is accurately described.

To determine an appropriate value for σ_{ABM}, we performed a sensitivity analysis across datasets. We evaluated performance using cross-boundary correctness and velocity confidence, two widely established metrics for RNA velocity evaluation. We found that velocity confidence remained high across the range of tested values (Appendix Figure S20b), while cross-boundary direction was also generally similar but showed slightly better performance at σ_{ABM} = 0.7 across datasets (Appendix Figure S20a). We have now included σ_{ABM} = 0.7 as the default value in the hyperparameter section of the Methods.

Regarding the neighborhood radius r, we selected r separately for each dataset such that each cell had, on average, approximately 15-20 spatial neighbors. We believe this is a reasonable choice because it provides enough local context to capture meaningful neighborhood effects while avoiding overly large neighborhoods that could blur local spatial structure or mix cells from distinct microenvironments. This strategy also makes the choice of r adaptable across different spatial transcriptomics technologies and spatial scales. We have added this explanation to the hyperparameter section of the Methods as well.

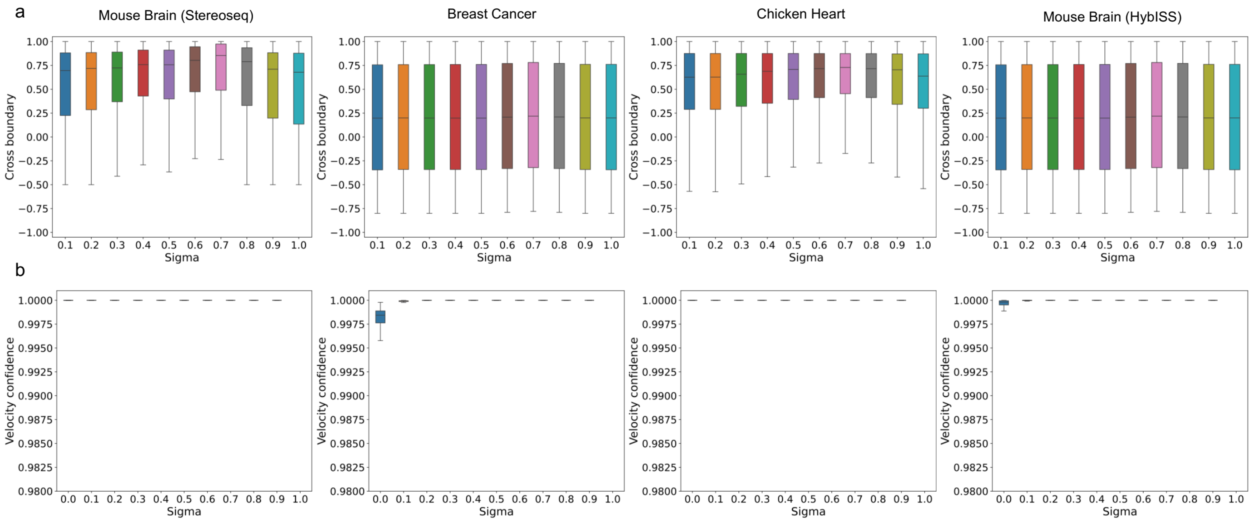

Appendix Figure S20: Quantitative performance of veloAgent across different σ_{ABM} values. a, Cross-boundary scores for σ_{ABM} values ranging from 0-1 (inclusive). This metric measures the correctness of predicted transitions across annotated cluster boundaries based on ground-truth developmental labels. Higher scores reflect better agreement with known trajectories. **b,** Velocity confidence scores for σ_{ABM} values ranging from 0-1 (inclusive). This metric quantifies how well predicted velocities align with local gene expression structure. Higher scores indicate more reliable predictions. $\sigma_{ABM} = 0.7$ is the default value used in veloAgent. Error bars show interquartile range.

Comment 9: (Lines 691-696) The in silico perturbation procedure lacks detail. Please clarify: which parameters (e.g., transcription rate α) are modified, and which parts of the model remain active during re-computation (without retraining).

Response: You raise an important point regarding the clarity of the in silico perturbation procedure. The manuscript has been revised to more explicitly describe this process. In our setting, RNA velocity is inferred based on three gene-specific kinetic parameters: the transcription rate (α), the splicing rate (β), and the degradation rate (γ). These parameters are inferred by the neural network for each gene in each cell and govern the dynamics between unspliced and spliced mRNA, jointly determining the direction and magnitude of RNA velocity.

Specifically, in our setting, perturbation is applied to either the transcription rate parameter (α) or the splicing rate parameter (β), but not both simultaneously. In the main results, perturbations are applied to α (Fig. 5), while perturbations of $\beta$are presented in Appendix Figure S11.

Importantly, perturbation is performed at the level of individual genes. For a selected gene, the corresponding parameter is modified across all cells—for example, setting the transcription rate α of that gene to zero, while parameters of all other genes remain unchanged. When such a perturbation is applied, the remaining kinetic parameters for that gene (β and γ) are kept fixed. RNA velocity is then recomputed using the updated parameter values without retraining the model.

As a result of this perturbation, the inferred RNA velocities of cells are altered, reflecting the impact of modifying the regulatory contribution of the perturbed gene. These changes in velocity are then quantitatively evaluated to assess how the perturbation shifts cellular trajectories, enabling us to compute a therapeutic score that measures the extent to which the perturbation drives cells toward a desired (e.g., healthy or target) state.

Because the perturbation is applied after the neural network has already inferred the kinetic parameters (α, β, γ), the neural network is not used during the in silico perturbation procedure. These clarifications have been added to the revised manuscript in the Methods section under the subsection "In silico Perturbation."

References

Lopez R, Regier J, Cole MB, Jordan MI, Yosef N (2018) Deep generative modeling for single-cell transcriptomics. *Nature methods* 15: 1053-1058

Raghavan V, Zheng Y, Li Y, Ding J (2025) Harnessing agent-based frameworks in CellAgentChat to unravel cell–cell interactions from single-cell and spatial transcriptomics. *Genome Research* 35: 1646-1663

Szklarczyk D, Kirsch R, Koutrouli M, Nastou K, Mehryary F, Hachilif R, Gable AL, Fang T, Doncheva NT, Pyysalo S (2023) The STRING database in 2023: protein–protein association networks and functional enrichment analyses for any sequenced genome of interest. *Nucleic acids research* 51: D638-D646

2nd Apr 2026

Manuscript Number: MSB-2026-13571R
Title: Dissecting and steering cell dynamics using spatially-informed RNA velocity with veloAgent
Author: Jun Ding
Vishvak Raghavan
Brent Yoon
Gregory Fonseca
Yue Li

Dear Dr Ding,

Thank you again for submitting your work to Molecular Systems Biology. We have now received the enclosed reports from three reviewers who kindly agreed to re-assess your work. As you will see below, all reviewers are satisfied with the revisions and are supportive of publication. I am therefore pleased to inform you that we are able to accept your manuscript, pending the following editorial-level amendments:

1. Please remove all figures from the manuscript file. Only the figure legends should remain at the end of the manuscript.

2. Please ensure that the funding information listed in the manuscript file is consistent with what is entered in the submission system. The following funders are missing from the submission system and need to be added: " Meakins-Christie Chair in Respiratory Research [to J.D.]; V.R received support from the Canada First Research Excellence Fund and the Fonds de recherche du Quebec awarded to the D2R Initiative at McGill University. This research was enabled in part by support provided by Calcul Quebec (calculquebec.ca) and the Digital Research Alliance of Canada (alliancecan.ca)".

3. Add missing callout sfor Appendix Table S2 and Figure 4c,f, Figure 6b.

4. Appendix:
- Please provide the Aappendix as a PDF instead of a DOCX.
- The appendix title page should read: "Appendix for '[Manuscript Title]'".
- The quality of the appendix figures is too low; please provide high-resolution figures.
- Appendix Figure S8: Panel E is not cited in the caption; please update the caption to include it.
- Appendix Figure S11: Ensure that the figure panels correspond to the panels cited in the caption - panel F is currently missing in the figure.
- Appendix Figure S16: Panels A and B need to be clearly indicated in the figure.

5. Please address the following issues in figure legends:
- Please note that the box plots need to be defined in terms of minima, maxima, centre and percentile in the legends of figures 2I-K; 3G, 6C-F
- Please note that information related to n is missing in the legends of figures 2I-K; 3G, 6C-F

6. Please remove the synopsis text and image from the manuscript file; they should be uploaded as separate files. The synopsis image is a bit blurry, please provide an updated version with better quality. Also, we recommend removing the VeloAgent spy/agent icon.

7. "DISCLOSURE STATEMENT AND COMPETING INTERESTS" should be renamed to "Disclosure and Competing Interests Statement".

8. Author checklist: The section "DNA and RNA sequences" is currently empty and needs to be completed.

9. The manuscript sections should be in the following order: Title page - Abstract - Introduction - Results - Discussion - Methods -Data Availability - Acknowledgments - Disclosure and Competing Interests Statement - References - Figure Legends - Expanded View Figure Legends

As a matter of course, please make sure that you have correctly followed the instructions for authors as given on the submission website.

Thank you for submitting this interesting paper to Molecular Systems Biology.

Kind regards,
Jingyi

Jingyi Hou, PhD
Senior Editor
Molecular Systems Biology

If you do choose to resubmit, please click on the link below to submit the revision online before 2nd May 2026.

https://msb.msubmit.net/cgi-bin/main.plex

*** PLEASE NOTE *** As part of the EMBO Press transparent editorial process initiative (see our Editorial at https://dx.doi.org/10.1038/msb.2010.72 , Molecular Systems Biology will publish online a Review Process File to accompany accepted manuscripts. When preparing your letter of response, please be aware that in the event of acceptance, your cover letter/point-by-point document will be included as part of this File, which will be available to the scientific community. More information about this initiative is available in our Instructions to Authors. If you have any questions about this initiative, please contact the editorial office (msb@embo.org).

Reviewer #1:

Well done.

Reviewer #2:

The authors did an excellent job addressing all of my concerns. I have no further comments.

Reviewer #3:

The authors have revised the manuscript very well. All previously raised concerns have been satisfactorily addressed, and I have no further questions or comments.

All minor editorial requests have been addressed by the authors.

16th Apr 2026
Manuscript number: MSB-2026-13571RR
Title: Dissecting and steering cell dynamics using spatially-informed RNA velocity with veloAgent

Dear Dr Ding,

Thank you again for sending us your revised manuscript. We are now satisfied with the modifications made and I am pleased to inform you that your paper has been accepted for publication.

Your manuscript will be processed for publication by EMBO Press. It will be copy edited and you will receive page proofs prior to publication. Please note that you will be contacted by Springer Nature Author Services to complete licensing and payment information.

You may qualify for financial assistance for your publication charges - either via a Springer Nature fully open access agreement or an EMBO initiative. Check your eligibility: https://link.springer.com/journal/44320/how-to-publish-with-us

Should you be planning a Press Release on your article, please get in contact with embo_production@springernature.com as early as possible in order to coordinate publication and release dates.

If you have any questions, please do not hesitate to contact the Editorial Office. Thank you for your contribution to Molecular Systems Biology.

Kind regards,
Jingyi

Jingyi Hou, PhD
Senior Editor
Molecular Systems Biology

>>> Please note that it is Molecular Systems Biology policy for the transcript of the editorial process (containing referee reports and your response letter) to be published as an online supplement to each paper. If you do NOT want this, you will need to inform the Editorial Office via email immediately. More information is available here: https://link.springer.com/partners/embo-press/editorial-policies#Peer%20review

EMBO Press Author Checklist

Corresponding Author Name: Jun Ding	
Journal Submitted to: Molecular Systems Biology	
Manuscript Number: MSB-2026-13571-T	

USEFUL LINKS FOR COMPLETING THIS FORM
The EMBO Journal - Author Guidelines
EMBO Reports - Author Guidelines
Molecular Systems Biology - Author Guidelines
EMBO Molecular Medicine - Author Guidelines

Reporting Checklist for Life Science Articles (updated January
This checklist is adapted from Materials Design Analysis Reporting (MDAR) Checklist for Authors. MDAR establishes a minimum set of requirements in transparent reporting in the life sciences (see Statement of Task: 10.31222/osf.io/9sm4x). Please follow the journal's guidelines in preparing your manuscript.
Please note that a copy of this checklist will be published alongside your article.

Abridged guidelines for figures
1. Data
The data shown in figures should satisfy the following conditions:
- the data were obtained and processed according to the field's best practice and are presented to reflect the results of the experiments in an accurate and unbiased manner.
- ideally, figure panels should include only measurements that are directly comparable to each other and obtained with the same assay.
- plots include clearly labeled error bars for independent experiments and sample sizes. Unless justified, error bars should not be shown for technical
- if n<5, the individual data points from each experiment should be plotted. Any statistical test employed should be justified.
- Source Data should be included to report the data underlying figures according to the guidelines set out in the authorship guidelines on Data

2. Captions
Each figure caption should contain the following information, for each panel where they are relevant:
- a specification of the experimental system investigated (eg cell line, species name).
- the assay(s) and method(s) used to carry out the reported observations and measurements.
- an explicit mention of the biological and chemical entity(ies) that are being measured.
- an explicit mention of the biological and chemical entity(ies) that are altered/varied/perturbed in a controlled manner.
- the exact sample size (n) for each experimental group/condition, given as a number, not a range;
- a description of the sample collection allowing the reader to understand whether the samples represent technical or biological replicates (including how many animals, litters, cultures, etc.).
- a statement of how many times the experiment shown was independently replicated in the laboratory.
- definitions of statistical methods and measures:
  - common tests, such as t-test (please specify whether paired vs. unpaired), simple χ2 tests, Wilcoxon and Mann-Whitney tests, can be unambiguously identified by name only, but more complex techniques should be described in the methods section;
  - are tests one-sided or two-sided?
  - are there adjustments for multiple comparisons?
  - exact statistical test results, e.g., P values = x but not P values < x;
  - definition of 'center values' as median or average;
  - definition of error bars as s.d. or s.e.m.

Please complete ALL of the questions below.
Select "Not Applicable" only when the requested information is not relevant for your study.

Materials

Newly Created Materials	Information included in the manuscript?	In which section is the information available? (Reagents and Tools Table, Materials and Methods, Figures, Data Availability Section)
New materials and reagents need to be available; do any restrictions apply?	Not Applicable	

Antibodies	Information included in the manuscript?	In which section is the information available? (Reagents and Tools Table, Materials and Methods, Figures, Data Availability Section)
For **antibodies** provide the following information:<br>- Commercial antibodies: RRID (if possible) or supplier name, catalogue number and or/clone number<br>- Non-commercial: RRID or citation	Not Applicable	

DNA and RNA sequences	Information included in the manuscript?	In which section is the information available? (Reagents and Tools Table, Materials and Methods, Figures, Data Availability Section)
**Short novel DNA or RNA including primers, probes**: provide the sequences.	Not Applicable	

Cell materials	Information included in the manuscript?	In which section is the information available? (Reagents and Tools Table, Materials and Methods, Figures, Data Availability Section)
**Cell lines:** Provide species information, strain. Provide accession number in repository **OR** supplier name, catalog number, clone number, and/**OR** RRID.	Not Applicable	
**Primary cultures:** Provide species, strain, sex of origin, genetic modification status.	Not Applicable	
Report if the cell lines were recently **authenticated** (e.g., by STR profiling) and tested for mycoplasma contamination.	Not Applicable	

Experimental animals	Information included in the manuscript?	In which section is the information available? (Reagents and Tools Table, Materials and Methods, Figures, Data Availability Section)
**Laboratory animals or Model organisms:** Provide species, strain, sex, age, genetic modification status. Provide accession number in repository **OR** supplier name, catalog number, clone number, **OR** RRID.	Not Applicable	
**Animal observed in or captured from the field:** Provide species, sex, and age where possible.	Not Applicable	
Please detail **housing and husbandry conditions**.	Not Applicable	

Plants and microbes	Information included in the manuscript?	In which section is the information available? (Reagents and Tools Table, Materials and Methods, Figures, Data Availability Section)
**Plants:** provide species and strain, ecotype and cultivar where relevant, unique accession number if available, and source (including location for collected wild specimens).	Not Applicable	
**Microbes:** provide species and strain, unique accession number if available, and source.	Not Applicable	

Human research participants	Information included in the manuscript?	In which section is the information available? (Reagents and Tools Table, Materials and Methods, Figures, Data Availability Section)
If collected and within the bounds of privacy constraints report on age, sex and gender or ethnicity for all study participants.	Not Applicable	

Core facilities	Information included in the manuscript?	In which section is the information available? (Reagents and Tools Table, Materials and Methods, Figures, Data Availability Section)
If your work benefited from core facilities, was their service mentioned in the acknowledgments section?	Not Applicable	

Design

Study protocol	Information included in the manuscript?	In which section is the information available? (Reagents and Tools Table, Materials and Methods, Figures, Data Availability Section)
If study protocol has been **pre-registered, provide DOI in the manuscript**. For clinical trials, provide the trial registration number **OR** cite DOI.	Not Applicable	
Report the **clinical trial registration number** (at ClinicalTrials.gov or equivalent), where applicable.	Not Applicable	

Laboratory protocol	Information included in the manuscript?	In which section is the information available? (Reagents and Tools Table, Materials and Methods, Figures, Data Availability Section)
Provide DOI OR other citation details if **external detailed step-by-step protocols** are available.	Not Applicable	

Experimental study design and statistics	Information included in the manuscript?	In which section is the information available? (Reagents and Tools Table, Materials and Methods, Figures, Data Availability Section)
Include a statement about **sample size** estimate even if no statistical methods were used.	Not Applicable	
Were any steps taken to minimize the effects of subjective bias when allocating animals/samples to treatment (e.g. **randomization procedure**)? If yes, have they been described?	Not Applicable	
Include a statement about **blinding** even if no blinding was done.	Not Applicable	
Describe **inclusion/exclusion criteria** if samples or animals were excluded from the analysis. Were the criteria pre-established? If sample or data points were omitted from analysis, report if this was due to attrition or intentional exclusion and provide justification.	Not Applicable	
For every figure, are **statistical tests** justified as appropriate? Do the data meet the assumptions of the tests (e.g., normal distribution)? Describe any methods used to assess it. Is there an estimate of variation within each group of data? Is the variance similar between the groups that are being statistically compared?	Yes	Figures

Sample definition and in-laboratory replication	Information included in the manuscript?	In which section is the information available? (Reagents and Tools Table, Materials and Methods, Figures, Data Availability Section)
In the figure legends: state number of times the experiment was **replicated** in laboratory.	Not Applicable	
In the figure legends: define whether data describe **technical or biological replicates**.	Not Applicable	

Ethics

Ethics	Information included in the manuscript?	In which section is the information available? (Reagents and Tools Table, Materials and Methods, Figures, Data Availability Section)
Studies involving **human participants**: State details of **authority granting ethics approval** (IRB or equivalent committee(s), provide reference number for approval.	Not Applicable	
Studies involving **human participants**: Include a statement confirming that **informed consent** was obtained from all subjects and that the experiments conformed to the principles set out in the WMA Declaration of Helsinki and the Department of Health and Human Services Belmont Report.	Not Applicable	
Studies involving **human participants:** For publication of **patient photos**, include a statement confirming that consent to publish was obtained.	Not Applicable	
Studies involving experimental **animals**: State details of **authority granting ethics approva**l (IRB or equivalent committee(s), provide reference number for approval. Include a statement of compliance with ethical regulations.	Not Applicable	
Studies involving **specimen and field samples:** State if relevant **permits** obtained, provide details of authority approving study; if none were required, explain why.	Not Applicable	

Dual Use Research of Concern (DURC)	Information included in the manuscript?	In which section is the information available? (Reagents and Tools Table, Materials and Methods, Figures, Data Availability Section)
Could your study fall under dual use research restrictions? Please check biosecurity documents and list of **select agents and toxins** (CDC): https://www.selectagents.gov/sat/list.htm	Not Applicable	
If you used a select agent, is the security level of the lab appropriate and reported in the manuscript?	Not Applicable	
If a study is subject to dual use research of concern regulations, is the name of the **authority granting approval and reference number** for the regulatory approval provided in the manuscript?	Not Applicable	

Reporting

The MDAR framework recommends adoption of discipline-specific guidelines, established and endorsed through community initiatives. Journals have their own policy about requiring specific guidelines and recommendations to complement MDAR.

Adherence to community standards	Information included in the manuscript?	In which section is the information available? (Reagents and Tools Table, Materials and Methods, Figures, Data Availability Section)
State if relevant guidelines or checklists (e.g., **ICMJE, MIBBI, ARRIVE, PRISMA**) have been followed or provided.	Not Applicable	
For **tumor marker prognostic studies,** we recommend that you follow the **REMARK** reporting guidelines (see link list at top right). See author guidelines, under 'Reporting Guidelines'. Please confirm you have followed these guidelines.	Not Applicable	
For **phase II and III randomized controlled trials**, please refer to the **CONSORT** flow diagram (see link list at top right) and submit the CONSORT checklist (see link list at top right) with your submission. See author guidelines, under 'Reporting Guidelines'. Please confirm you have submitted this list.	Not Applicable	

Data Availability

Data availability	Information included in the manuscript?	In which section is the information available? (Reagents and Tools Table, Materials and Methods, Figures, Data Availability Section)
Have **primary datasets** been deposited according to the journal's guidelines (see 'Data Deposition' section) and the respective accession numbers provided in the Data Availability Section?	Not Applicable	
Were **human clinical and genomic datasets** deposited in a public access-controlled repository in accordance to ethical obligations to the patients and to the applicable consent agreement?	Not Applicable	
Are **computational models** that are central and integral to a study available without restrictions in a machine-readable form? Were the relevant accession numbers or links provided?	Yes	Data Availability
If publicly available data were reused, provide the respective **data citations in the reference list**.	Yes	Data Availability
